# Structural, Biochemical, and Computational Characterization of Sulfamides as Bimetallic Peptidase Inhibitors

**DOI:** 10.1021/acs.jcim.3c01542

**Published:** 2024-01-15

**Authors:** Zora Novakova, Zahra Aliakbar Tehrani, Radek Jurok, Lucia Motlova, Zsofia Kutil, Jiri Pavlicek, Shivam Shukla, Cindy J. Choy, Barbora Havlinova, Petra Baranova, Clifford E. Berkman, Martin Kuchar, Jiri Cerny, Cyril Barinka

**Affiliations:** † Institute of Biotechnology of the Czech Academy of Sciences, BIOCEV, Prumyslova 595, 252 50 Vestec, Czech Republic; ‡ Forensic Laboratory of Biologically Active Substances, University of Chemistry and Technology Prague, Technická 3, 166 28 Prague 6, Czech Republic; § Department of Chemistry, Washington State University, Pullman, Washington 99163, United States

## Abstract

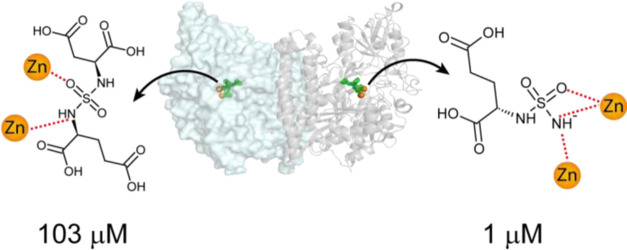

The sulfonamide function is used extensively as a general building block in various inhibitory scaffolds and, more specifically, as a zinc-binding group (ZBG) of metalloenzyme inhibitors. Here, we provide biochemical, structural, and computational characterization of a metallopeptidase in complex with inhibitors, where the mono- and bisubstituted sulfamide functions are designed to directly engage zinc ions of a bimetallic enzyme site. Structural data showed that while monosubstituted sulfamides coordinate active-site zinc ions via the free negatively charged amino group in a canonical manner, their bisubstituted counterparts adopt an atypical binding pattern divergent from expected positioning of corresponding tetrahedral reaction intermediates. Accompanying quantum mechanics calculations revealed that electroneutrality of the sulfamide function is a major factor contributing to the markedly lower potency of bisubstituted compounds by considerably lowering their interaction energy with the enzyme. Overall, while bisubstituted uncharged sulfamide functions can bolster favorable pharmacological properties of a given inhibitor, their use as ZBGs in metalloenzyme inhibitors might be less advantageous due to their suboptimal metal–ligand properties.

## Introduction

Sulfonyl and sulfamide functionalities belong to the most ubiquitous building blocks in medicinal bioactive compounds, including antibiotics, anti-inflammatory, antitumor, antidiabetic, antiviral, and anticonvulsant agents.^1^ In fact, over 150 drugs containing one of the two moieties (and their derivatives) have been approved by the FDA. In addition to the ease of synthesis/derivatization, favorable properties of sulfamides include their resistance to reduction at the sulfur center, stability against hydrolysis, and high polarizability, yet a net neutral charge, that could contribute to enhanced water solubility and bioavailability of the sulfamide-based drugs.^2^ More specifically, the sulfamide function can also serve as a zinc-binding group (ZBG) in the design of inhibitors targeting selected metallohydrolases. Among these, the best studied are inhibitors of carbonic anhydrases (CA), where the ionized NH^–^ sulfamide group coordinates the active-site zinc ion and thus impairs the formation of the zinc hydroxide nucleophile critical for the hydration of CO_2_ to bicarbonate.^2−6^ Similarly, Park and colleagues identified sulfamide-based compounds as a novel type of transition state analogues of carboxypeptidase A (CPA), where the sulfamide moiety directly engages the catalytic zinc ion and displaces the catalytic water molecule/hydroxide anion required for substrate hydrolysis.^7^

Prostate-specific membrane antigen (PSMA), also known as glutamate carboxypeptidase II (GCPII), folate hydrolase (FOLH1), or N-acetylated α-linked acidic dipeptidase (NAALADase), is a membrane-bound metallopeptidase, featuring two zinc ions in its active site. In healthy human tissues, the prominent sites of PSMA expression include the nervous system, kidneys, salivary glands, proximal small intestine, and prostate. Importantly, the PSMA is overexpressed on prostate cancer cells and the enzyme thus attracts significant attention as a target for the delivery of imaging and therapeutic agents and continues to serve as an important biomarker of prostate cancer.^8^ Furthermore, the inhibition of PSMA in the nervous system has been shown to attenuate neurotoxicity associated with enhanced glutamate transmission and PSMA-specific inhibitors have demonstrated efficacy in multiple preclinical animal models, including neuropathic and inflammatory pain, traumatic brain injury, stroke, and amyotrophic lateral sclerosis.^9,10^

Within the last two decades, several classes of potent PSMA inhibitors have been reported in the literature. Prototypical PSMA-specific ligands comprise a glutamate/glutarate moiety attached to a zinc-binding group, and their productive combination ensures high affinity and selectivity of the resulting inhibitor. Hydroxamate, phosphinate/phosphoramidate, and thiol functionalities serve as the traditional ZBGs of glutamate-derived PSMA inhibitors. In more complex compounds derived from the NAAG dipeptide, urea- and phosphorus-based ZBGs that mimic the planar peptide bond and the gem-diolate transition state complex, respectively, are the most frequently used and advanced into the clinic.^9,11,12^ While both these inhibitor classes have very high affinity with subnanomolar inhibition constants, phosphorus-based inhibitors can suffer from poor oral bioavailability and rapid renal clearance, which may limit their practical value as a clinical therapeutic agent, necessitating synthesis of ester-type prodrugs.^13^ Substitution of the highly charged phosphorus-based functionality by a neutral sulfamide such as the ZBG in PSMA-specific inhibitors could, in theory, remedy some of the above-mentioned shortcomings. However, despite being promoted as the proficient ZBG in the case of CA-IX and CPA, sulfamide-based PSMA inhibitors lack the potency of their phosphorus-based counterparts.^14,15^

To identify molecular mechanisms behind markedly different potencies of bisubstituted sulfamides and phosphinates/phosphonates, we solved X-ray structures of complexes between PSMA, a well-studied bimetallic metallopeptidase, and sulfamide-based inhibitors. Complemented by quantum mechanics (QM) cluster model calculations and compared to structurally matching phosphinates, our data reveal that electroneutrality of the sulfamide function is a major factor contributing to the markedly lower potency of bisubstituted compounds by considerably lowering their interaction energy with the enzyme. These findings suggest that suboptimal metal-binding properties of bisubstituted sulfamides shall be cautiously evaluated when designing high-affinity metalloprotein inhibitors as this aspect can represent a liability under circumstances, where the sulfamide function is designed to directly coordinate a metal ion of a target metal-dependent enzyme.

## Results

### Inhibitor Synthesis and Potency

**Scheme 1:**
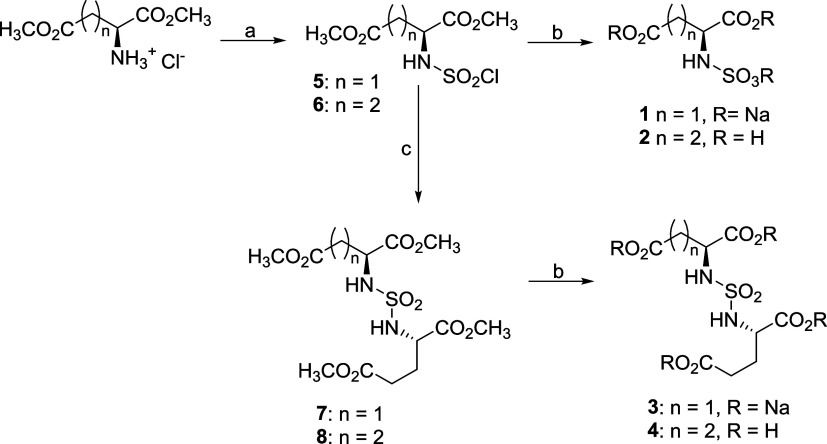
Reagents and Conditions: (a) SO_2_Cl_2_, CH_3_CN, 60 °C, 24 h, 77–80%; (b) (i) LiOH·H_2_O, CH_3_OH/THF; (ii) HCl; and (iii) NaHCO_3_ n = 1, 65–79%; (c) (*S*)-Dimethyl Glutamate Hydrochloride, DCM, TEA, 0 °C, 72–81%

A simple method amenable to parallel synthesis was employed to synthesize sulfamic acids **1** and **2** and sulfamides **3** and **4** (Scheme 1). Briefly, commercially available amino acid methyl esters were treated with sulfuryldichloride in acetonitrile to provide the corresponding sulfamoyl chlorides **5** and **6.**(16) The resulting chlorides were then treated with lithium hydroxide in methanol to provide sulfamic acids **1** and **2**, whereas the acid **1** was preferably isolated as a trisodium salt. Nucleophilic displacement of the chlorine in **5** and **6** was carried out with (*S*)-dimethyl glutamate to generate methyl-protected sulfamides **7** and **8**. Finally, **7** and **8** were deprotected by ester hydrolysis to form sulfamides **3** and **4**. It is interesting to note that the original oxazolidinone protocol for the synthesis of **3** and **4**(15,17) yielded, in our hands, a mixture of diastereomers, instead of expected pure enantiomeric products. We suspect that the observed racemization was a consequence of prolonged heating in the presence of excess base. The monosubstituted glutamyl sulfamide **9** was prepared as described previously.^15^

**Figure 1:**
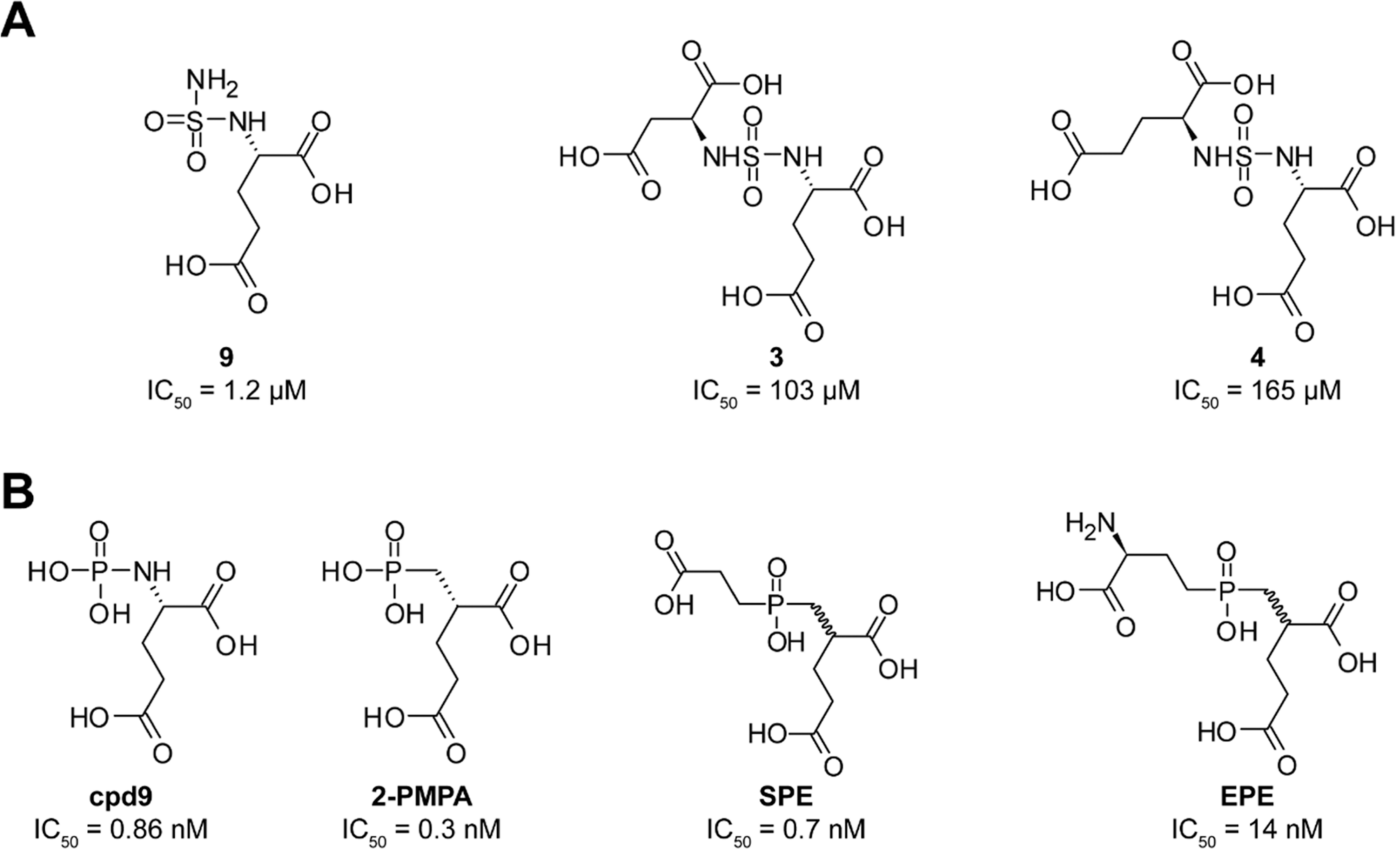
Formulas of sulfamides (A) and the corresponding phosphorus-based inhibitors (B) of PSMA. IC_50_ values for sulfamides were determined using a radioenzymatic assay with ^3^H-NAAG as a substrate and IC_50_ values for phosphorus-based compounds were taken from refs (18−20).

Inhibitory potency of the studied compounds (Figure 1) was determined using purified human recombinant PSMA together with *N*-acetyl-l-aspartyl-[^3^H]-l-glutamate as a substrate. Compound **9** was the most potent PSMA inhibitor with IC_50_ = 1.2 μM. The N^1^ functionalization of **9** has a significant negative impact on the inhibition potency, resulting in IC_50_ values of 103 and 165 μM for **3** and **4**, respectively.

### Structural and Computational Characterization of the PSMA/**9** Complex

The monosubstituted glutamyl sulfamide **9** is an isostere of 2-(phosphonomethyl) pentanedioic acid (2-PMPA) and **cpd9** inhibitors with reported IC_50_ values of 0.3 and 0.86 nM against human PSMA, respectively^19,20^ (Figure 1). To identify attributes responsible for over 1000-fold lower affinity of **9** compared to phosphorus isosteres, we determined the X-ray structure of the PSMA/**9** complex (PDB code: 4W9Y; Table 1) and complemented the structural data with QM cluster model calculations.

**Table 1:** Data Collection and Refinement Statistics^a^

data collection statistics			
inhibitor	**9**	**3**	**4**
PDB code	4W9Y	6SKH	6SGP
space group	*I*222	*I*222	*I*222
unit-cell parameters *a, b, c* (Å)	101.2, 130.4, 158.5	101.6, 130.5, 159.2	101.3, 130.4, 158.5
wavelength (Å)	0.92	1.03	1.03
resolution limits (Å)	50.0–1.64 (1.73–1.64)	50.00–1.58 (1.61–1.58)	50.00–1.58 (1.67–1.58)
no. of unique refl.	94,692 (15617)	143,110 (29834)	141,047 (22618)
redundancy	5.8 (5.8)	4.4 (4.3)	4.4 (4.9)
completeness (%)	99.2 (95.7)	99.2 (97.8)	97 (97.3)
CC 1/2 (%)	99.9 (88.8)	99.9 (86.0)	100.0 (83.0)
*I*/σ(*I*)	19.0 (2.5)	19.5 (2.5)	18.50 (1.97)
*R*_merge_	0.066 (0.820)	0.033 (0.528)	0.040 (0.689)

refinement statistics			
resolution limits (Å)	28.43–1.64 (1.68–1.64)	49.21–1.58 (1.62–1.58)	46.9–1.58 (1.62–1.58)
total number of reflections	121,368 (9347)	136,083 (10,412)	134,096 (10,228)
number of reflections in working set	114,980 (8879)	132,057(9899)	127,002(9735)
number of reflections in test set	6388 (468)	7026 (513)	7094(493)
*R*/*R*_free_ (%)	15.9/17.7 (26.0/28.4)	17.0/19.1 (24/25.2)	18.0/20.1 (33.4/34.1)
total number of non-H atoms	6524	7027	6561
number of non-H protein atoms	5928	6136	5838
number of inhibitor molecules	1	1	1
number of water molecules	582	591	434
average *B*-factor (Å^2^)	26.9	34.9	30.6
protein	26.1	33.3	30.5
water molecules	35.8	30.9	41.0
inhibitor	15.6	42.3	37.9
bRamachandran plot (%)			
most favored	97.5	96	96
additionally allowed	2.3	4	4
disallowed	0.2 (Val382)		
RMS deviations: bond lengths (Å)	0.010	0.015	0.008
bond angles (deg)	1.54	1.58	1.35
chiral centers (Å^3^)	0.12	0.106	0.084

a Values in parentheses are for the highest resolution shells.

b Structures were analyzed using the MolProbity package.^24^

**Figure 2:**
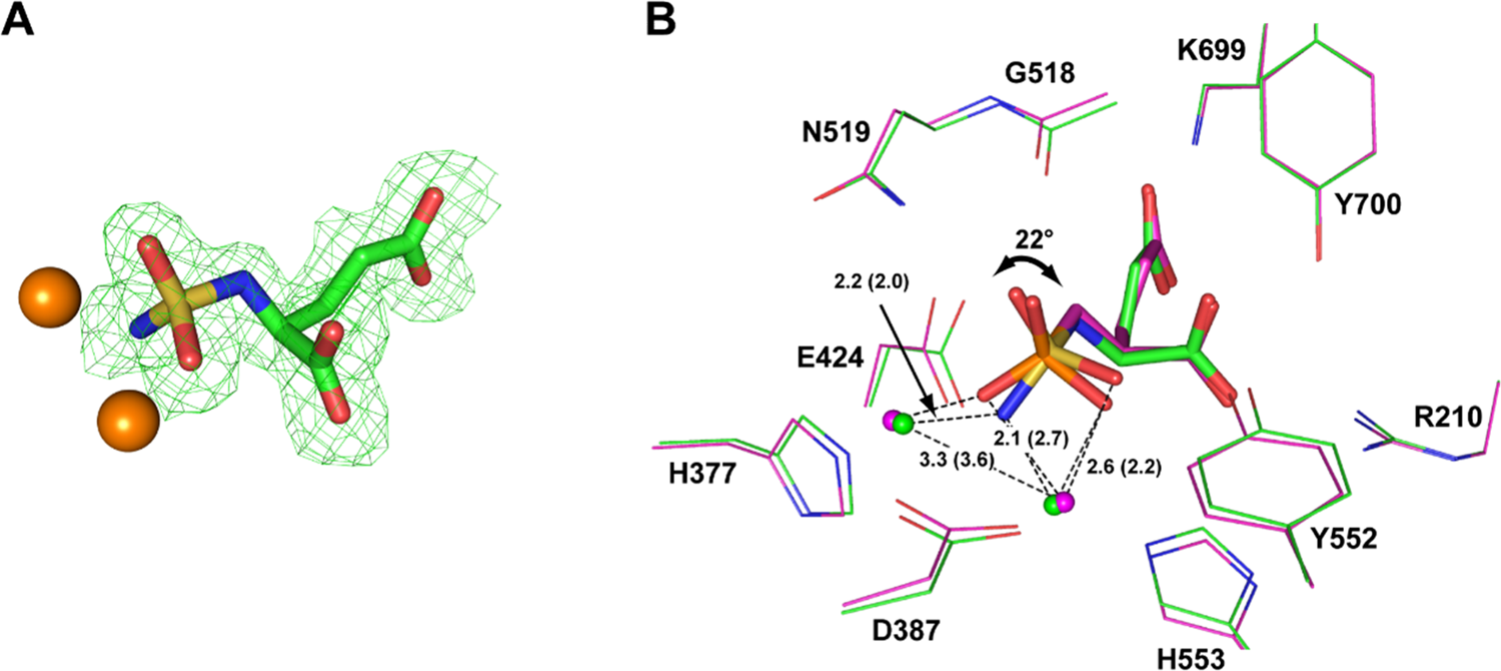
X-ray structure of the PSMA/**9** complex. (A) Compound **9** was modeled into the well-defined Fo-Fc positive density peaks contoured at the 3.0σ level. (B) Superposition of **9** (PDB code: 4W9Y; green carbon atoms and zinc ions) and **2-PMPA** (PDB code: 2PVW; magenta carbon atoms and zinc ions) in the active site of PSMA. While pentanedioic parts of both inhibitors overlap perfectly, there are differences in the alignment of their zinc-binding functionalities as the triangular base of the tetrahedral sulfamide headgroup is rotated by 22° compared to the **2-PMPA** phosphinate. Inhibitors are shown in stick representation and PSMA residues as lines. Distances of zinc-binding groups to zinc ions are in angstroms (dashed lines) with distances for the PSMA/**2-PMPA** complex shown in parentheses.

At the 1.64 Å resolution limit, the interpretable Fo-Fc electron density representing the active site-bound **9** was clearly observed in the internal PSMA pocket and the inhibitor was fitted into the density in the final stages of the refinement (Figure 2A). Positioning of the glutamate moiety of **9** in the S1′ site of PSMA conforms to the “canonical” binding mode observed previously for other PSMA-specific inhibitors and is virtually indistinguishable from the structure of the PSMA/2-PMPA complex (PDB code: 2PVW; Figure 2B). Surprisingly though, there are marked differences in the pattern of interaction of the zinc-binding groups with the active-site zinc ions. The sulfamide function is rotated by 22°, resulting in alterations in the coordination sphere of the active-site zinc ions. The amine group of the sulfamide is placed nearly equidistantly from both Zn^2+^ ions with interatomic distances 2.2 and 2.1 Å, respectively, “forcing” the oxygen atom into the second coordination shell of the catalytic Zn^2+^ at a distance of 2.6 Å. Furthermore, the distance between the zinc ions is shortened in the PSMA/**9** complex to 3.3 Å (from 3.6 Å in the PSMA/**2-PMPA** complex). The shorter inter-zinc distance is reminiscent of situations where the catalytic hydroxide ion is positioned in between the zincs, such as in PSMA complexes with free glutamate, urea-based inhibitors, and NAAG.^21−23^ The zinc relocation is accompanied by concomitant repositioning of zinc-coordinating residues (His377, Asp387, and His553; Figure 2B), underscoring the flexibility of the bimetallic active site of PSMA that is required for the substrate hydrolysis but, at the same time, can be exploited for the design of inhibitors with distinct ZBGs.

An ambiguity existed concerning the placement of the amino group and the two sulfonyl oxygen atoms within the electron density of the PSMA/**9** complex, as it is impossible to directly distinguish amino nitrogen and sulfonyl oxygen atoms attached to the central sulfur atom simply from the Fo-Fc difference map. However, both the analysis of the hydrogen bonding pattern between PSMA and the sulfamide function as well as QM cluster model calculations (see below) clearly support the model, in which the amino group is the primary zinc-interacting function. At this configuration, the first sulfonyl oxygen atom accepts a hydrogen bond from the amide group of Asn519 (2.9 Å), while the second oxygen atom is hydrogen-bonded to the hydroxyl group of the Tyr552 side chain (2.6 Å). Rotating the sulfamide functionality by ±120° would introduce unfavorable steric clashes with PSMA residues, and therefore, this possibility is much less likely.

**Figure 3:**
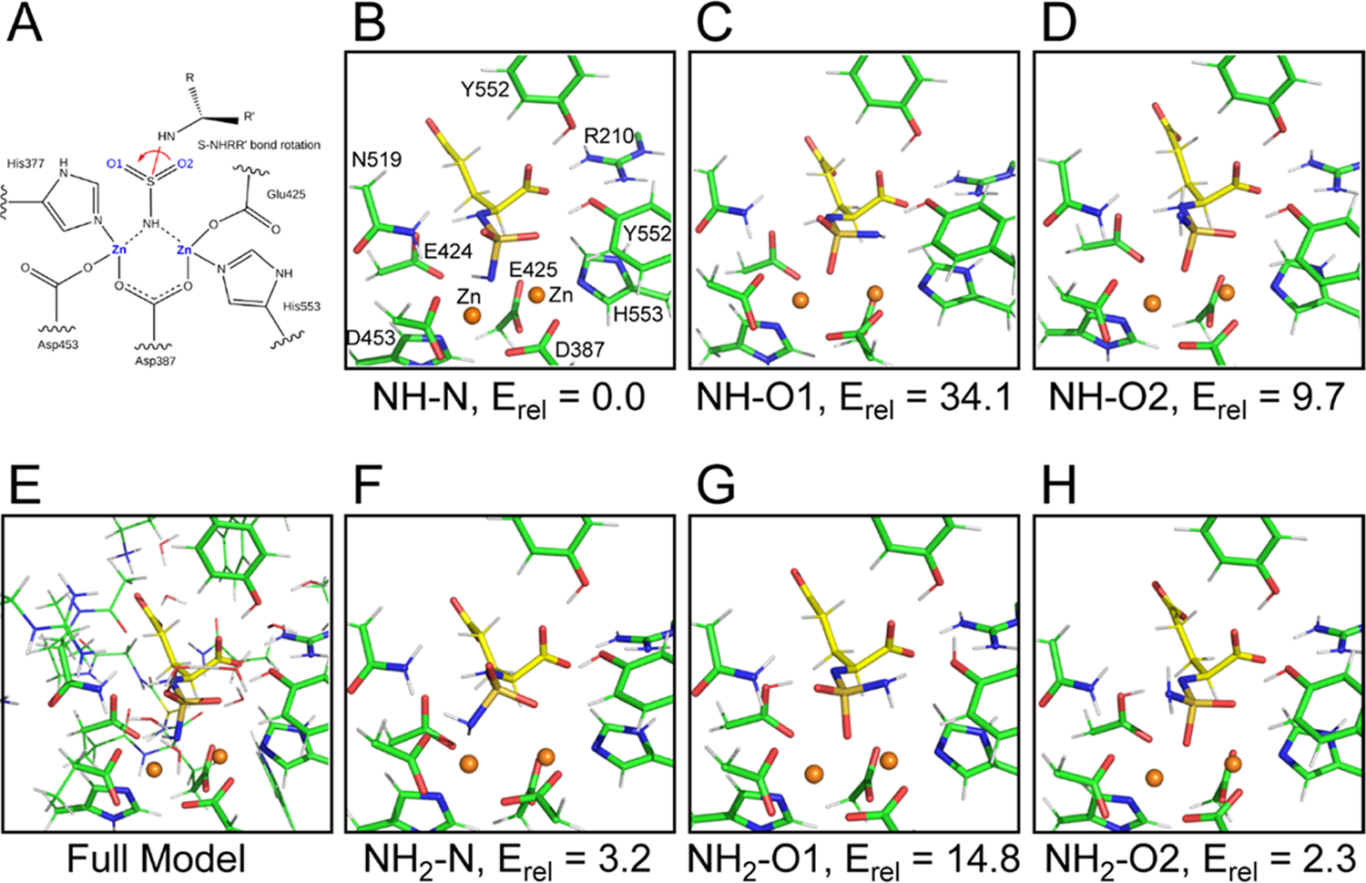
QM-optimized models of possible conformers of the PSMA/**9** complex obtained by rotation around the S–N bond of the inhibitor (A; red). The NH–N, NH–O1, and NH–O2 models (B–D) contain negatively charged NH^–^ groups, while the NH_2_–N, NH_2_–O1, and NH_2_–O2 models (F–H) correspond to neutral NH_2_ states. The inhibitor (yellow carbons) and selected PSMA residues (green carbons) are shown in stick representation, and zinc ions are shown as orange spheres. In the full model (E), the more distant PSMA residues and water molecules otherwise hidden for the sake of clarity are shown as lines. The relative energies for optimized models of the NH^–^ and NH_2_ configurations are shown in kcal/mol. Cartesian coordinates of the full structures are provided in the Supporting Information.

The structural data were next complemented by QM cluster model calculations to obtain more quantitative insights into the interactions of PSMA with **9**. To this end, we prepared models of six possible binding inhibitor configurations that are derived from the crystal structure by the ±120° rotation of the sulfamide functional group. The models also encompass both the negatively charged NH^–^ (NH–N, NH–O1, and NH–O2) and neutral NH_2_ (NH_2_–N, NH_2_–O1, and NH_2_–O2) protonation states of the terminal amino group (Figure 3), where the –N, –O1, and –O2 terms denote the atoms closest to the binuclear zinc center. All models were optimized at the DFT level and the total energy of the complex as well as the PSMA/sulfamide interaction energy was calculated at the DFT-D3/TPSS/def2-TZVP level with the water-COSMO model for solvation treatment. The calculated total energy of PSMA/sulfamide complexes shows the following order of relative energies: *E*_rel_: NH–N (0.0) > NH_2_–O2 (2.3) > NH_2_–N (3.2) > NH–O2 (9.7) > NH_2_–O1 (14.8) > NH–O1 (34.1) in kcal/mol, where NH–N and NH_2_–O2 models are the most stable conformers for the negatively charged NH^–^ and neutral NH_2_ models, respectively. Additionally, interaction energies (*E*_int_ = *E*(AB) – *E*(A) – *E*(B)) between PSMA and QM cluster models of **9** with different geometries and charges identified NH–N also as the strongest interacting variant, supporting the significant contribution of negatively charged sulfamide to the interaction with zinc ions. Overall, combined QM results corroborate the experimentally derived structure of the PSMA/**9** complex, where the amino group bridging active-site zinc ions bears the negative charge (NH^–^).

### Structural and Computational Characterization of Bisubstituted Sulfamides **3** and **4**

**Figure 4:**
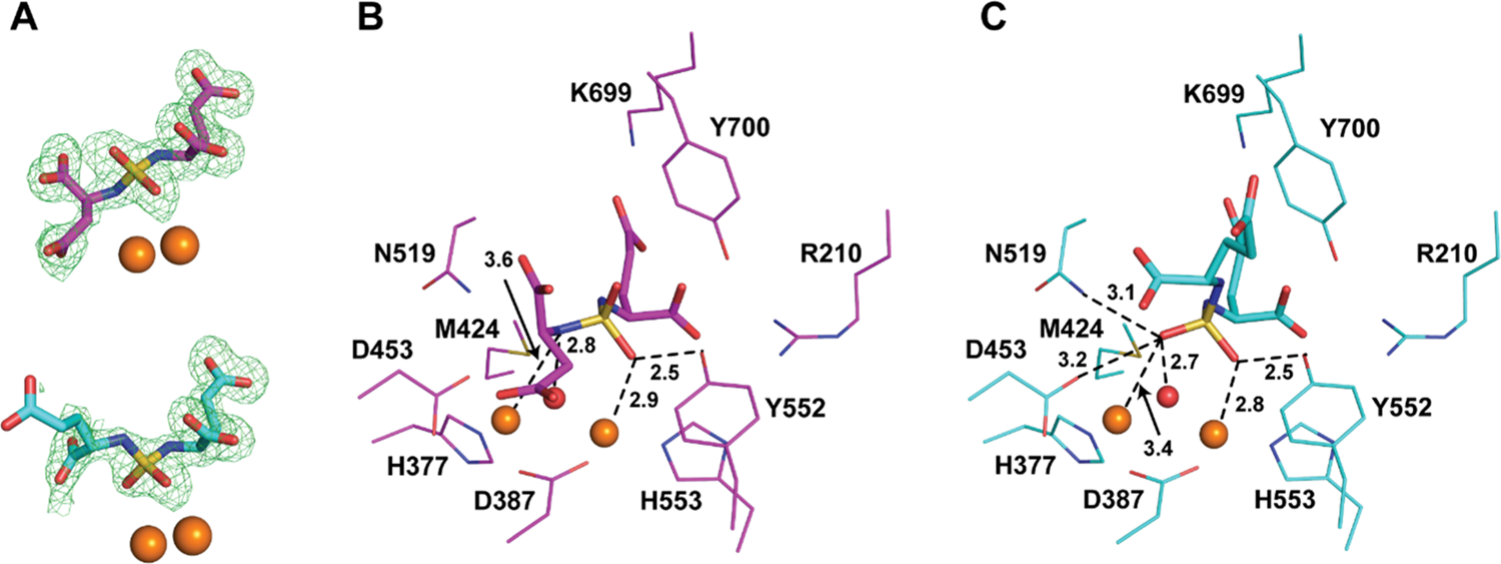
X-ray structures of the PSMA/**3** (PDB code: 6SKH) and PSMA/**4** complexes (PDB code: 6SGP). (A) The Fo-Fc omit map (green) is contoured at 3.0σ and inhibitors are shown in stick representation with atoms colored red (oxygen), blue (nitrogen), yellow (sulfur), magenta (carbons, compound **3**), and cyan (carbons, compound **4**). Zinc ions are shown as orange spheres. (B, C) Details of interactions are in the bimetallic active site of PSMA. PSMA residues are shown as lines, compounds **3** (B) and **4** (C) are in stick representation, and the activated water molecule/OH^–^ anion as a red sphere. Distances are given in angstroms (dashed lines).

X-ray structures of complexes between the inactive PSMA(E424M) mutant and bifunctional sulfamides **3** and **4** were both determined to the high-resolution limit of 1.58 Å (PDB code: 6SKH and 6SGP, respectively; Table 1). The use of the hydrolytically inactive PSMA(E424M) mutant was required due to the fact that upon cocrystallization of **3** and **4** with the wild-type enzyme, the inhibitors were hydrolyzed and only the hydrolytic products were observed in the PSMA active site (data not shown). Similar to the PSMA/**9** complex, the P1′ glutamates of **3** and **4** are well-defined in the Fo-Fc difference map and they occupy overlapping canonical positions within the S1′ pocket (Figure 4A). Interestingly, the placement of the sulfamide functionality differs between the two compounds. In the case of **3**, the O1 sulfamide oxygen is hydrogen-bonded to Tyr552 (2.5 Å) and coordinates (the second shell) the catalytic zinc with an interatomic distance of 2.9 Å. The P1 amide atom is H-bonded to the catalytic water/OH^–^ (2.8 Å) and in the distance of 3.6 Å from the cocatalytic zinc ion. In the PSMA/**4** complex, the sulfamide function is rotated by 120 degrees. Consequently, the positioning and interaction pattern of the first sulfamide oxygen is identical to that of **3**, while the second oxygen atom is rotated to the position of the P1 nitrogen atom of **4**, where it is hydrogen-bonded to the catalytic water/OH^–^ (2.7 Å), Asn519 (3.1 Å), and Asp453 (3.2 Å), and at a distance of 3.4 Å from the cocatalytic zinc ion. At the P1 part of the inhibitors, the α-carboxylate groups engage in ionic interactions with positively charged guanidinium groups of Arg534 and Arg536 comprising the arginine patch and H-bond with the amide of the Asn519 side chain. Finally, the β-carboxylate group of **3** forms ion pairs and the hydrogen bond with Arg536 (3.0 and 3.3 Å) and Ser454 (2.5 Å), respectively, while the γ-carboxylate of **4** is disordered in the structure pointing toward its positional flexibility and the absence of strong interactions with residues of the enzyme.

**Figure 5:**
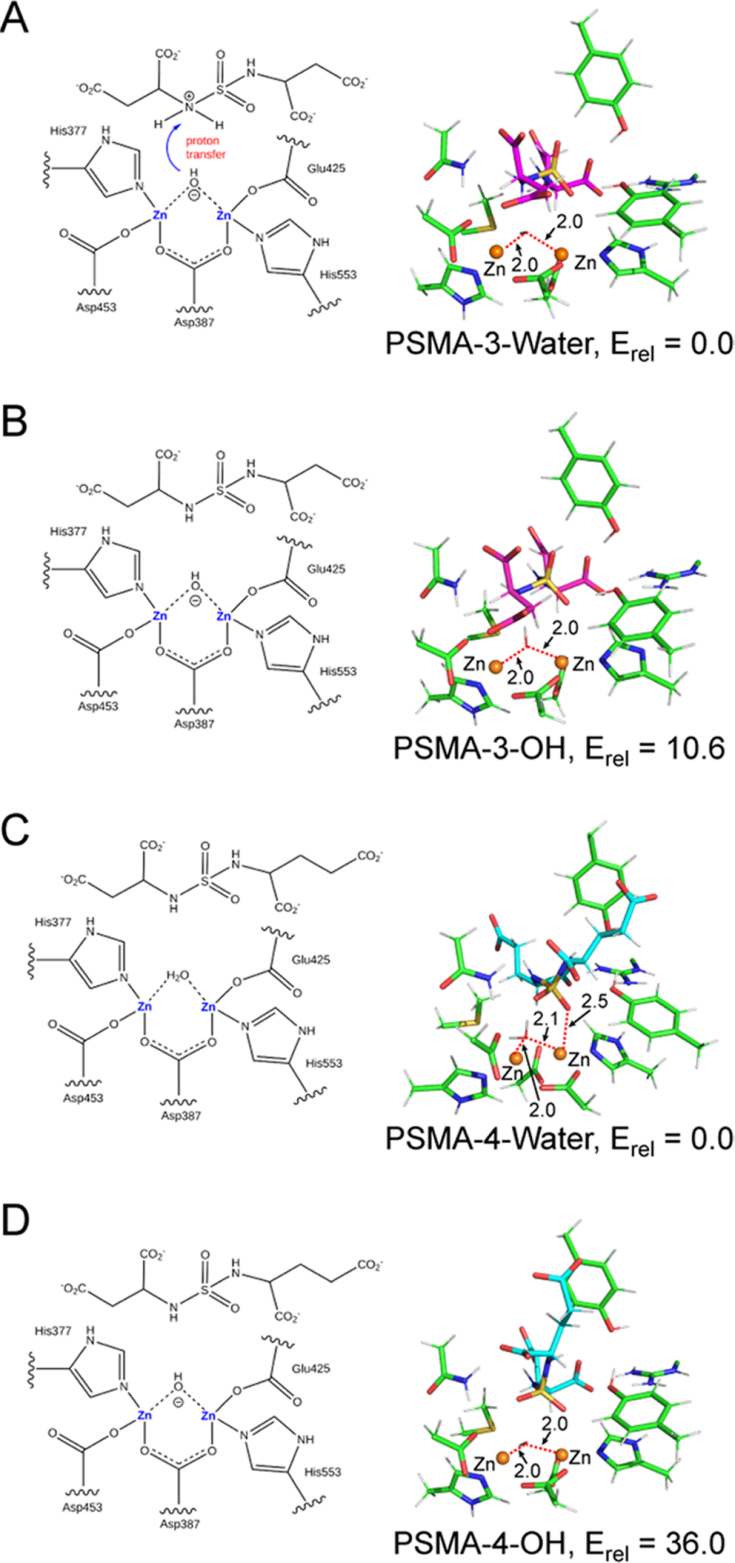
(A, B) QM-optimized structures of complexes of compound **3** were obtained with water and with OH^–^ in the active site of the mutated PSMA(E424M). All results were obtained at the DFT-D3/TPSS/def2-TZVP level with the water-COSMO model. (C, D) QM-optimized structures of complexes of compound **4** with water and with OH^–^ in the active site of the mutated PSMA(E424M). The inhibitor and selected surrounding residues are shown as sticks and zinc ions as orange spheres. The distances are shown in angstroms. Relative energies are reported in kcal/mol.

Accompanying QM calculations provided additional quantitative insights into energetics of PSMA interactions with the bisubstituted sulfamides **3** and **4**, including the protonation states of key interacting residues and the identity of the active site-bound water/hydroxide anion (Figure 5). The computational models’ setup, their geometry optimization, and energy calculations were carried out essentially, as described above for ligand **9**, and the QM region comprised 367 and 376 heavy atoms for complexes of **3** and **4**, respectively. In the more stable **3-Water** optimized structure (Figure 5A), the zinc interactions are formed by the water oxygen at comparable distances of about 2.0 Å. Interestingly, during the QM optimization, the activated bridging water molecule loses one of its protons to the neighboring –NH group of **3** forming a 1.6 Å long hydrogen bond between the deprotonated water and the protonated inhibitor as shown in Figure 5A. In the optimized **3-OH** structure, where the bridging water is replaced by an OH^–^ moiety (Figure 5B), the bridging OH^–^ interacts symmetrically with zinc ions at a distance of 2.0 Å and at the same time forms a longer (1.9 Å) hydrogen bond with the inhibitor. The above findings are in principle recapitulated for the QM-optimized PSMA/**4** structure, where the water complex (**4-Water**) is also more stable (by 36 kcal/mol) compared to the PSMA/**4-OH** complex (Figure 5C,D).

All reported energies are calculated using continuum solvation with the dielectric constant of bulk water (78.4). Although the ligand in the PSMA binding cavity is rather water accessible—on average, there are nearly 13 and over 14 water molecules within 3.5 Å from MD simulations of PSMA/**3-Water** and PSMA/**4-Water** complexes, respectively—the expected ε value would be lower. To assess the effect of ε value on the relative energies, we performed a series of single-point DFT-D3/TPSS/def2-TZVP calculations using different dielectric constants (ε = 4, 8, 20, and 40) with the results provided in Tables S1 and S2. The results show that the relative order of energies remains the same down to the dielectric constant value of 8 (the only change in the order was observed for the most extreme hydrophobic value of 4 but still the energy difference was rather small). The energy values were also comparable down to at least an epsilon of 20, showing that the conclusions are not affected by the initial choice of the epsilon value significantly.

It is interesting to note that the observed proton transfer between the water molecule and the amide nitrogen of the inhibitor in the **PSMA/3-Water** model could resemble an initial step of the experimentally observed hydrolysis of **3** in the active site of the wild-type enzyme during crystallization (data not shown). A similar mechanism is possible in the case PSMA/**4-Water;** however, this would require a larger scale rearrangement of **4** in the active site of PSMA. At the same time, given the expected conformational flexibility of **4** in the crystal structure (as witnessed by the weak Fo-Fc peaks for the P1 moiety of the inhibitor, see Figure 4), such a rearrangement can be plausible. A higher flexibility of **4** can also be observed from MD simulations. The more distant carboxylate and the atoms deeper in the binding cavity show larger fluctuations compared to ligand **3**. However, under the simulation conditions (3 × 100 ns for each ligand), both ligands remained bound in all of the simulated replicas and were only oscillating around the crystal structure positions without changing their conformations. The RMSF values for the ligand atoms are summarized in Figure S1.

## Discussion

Metalloenzymes, which require metal ions for their catalytic activity, play essential roles in a number of (patho)physiological processes, and their inhibition thus has become an effective strategy for the treatment of various pathologies. The vast majority of metalloenzyme inhibitors comprise a functional group that coordinates a catalytic metal center, and this interaction contributes prominently to high affinity and selectivity of a resulting inhibitor. Consequently, a wide array of metal-binding groups, including hydroxamates, sulfamides, heterocycles, thiols, and phosphonates, is employed for the design of metalloenzyme inhibitors to bolster and fine-tune their desired drug-like properties.^25,26^ Our data mining of the Protein Data Bank revealed that no structural information exists on binuclear metalloenzymes, where the sulfamide group of a small-molecule inhibitor would directly engage the bimetallic active site. Therefore, the structures reported here represent the first examples of such complexes and can serve as models for future studies aimed at the design of sulfamide-based inhibitors of binuclear metalloenzymes.

Similar to mononuclear metalloenzymes, a monosubstituted sulfamide coordinates PSMA active-site metal ions via its NH group, which is most likely in its negatively charged NH^–^ form.^27^ However, contrary to carbonic anhydrase, carboxypeptidase A or TAFI (thrombin activatable fibrinolysis inhibitor) inhibitors, where the primary sulfamide serves as an effective zinc-binding group,^2,4,7,28,29^ its incorporation into PSMA-targeting compounds is much less favorable. While the potency of compound **9** is approximately 350-fold higher compared to free glutamate, which is devoid of any ZBG, it is approximately 13-, 5-, 1400-, and 4000-fold less potent when compared to structurally matching inhibitors, comprising thiol, hydroxamate, phosphoramidate, and phosphinate ZBGs, respectively.^19,20,30−32^ Previous reports have demonstrated that the p*K*_a_ of the sulfamide NH can be modulated by incorporating electron-withdrawing groups adjacent to the nitrogen and that its negative charge contributes to the higher affinity of sulfamides for metalloproteases.^27,33,34^ Future studies could thus exploit this electron-withdrawing strategy to design more potent PSMA-inhibiting sulfamides.

**Figure 6:**
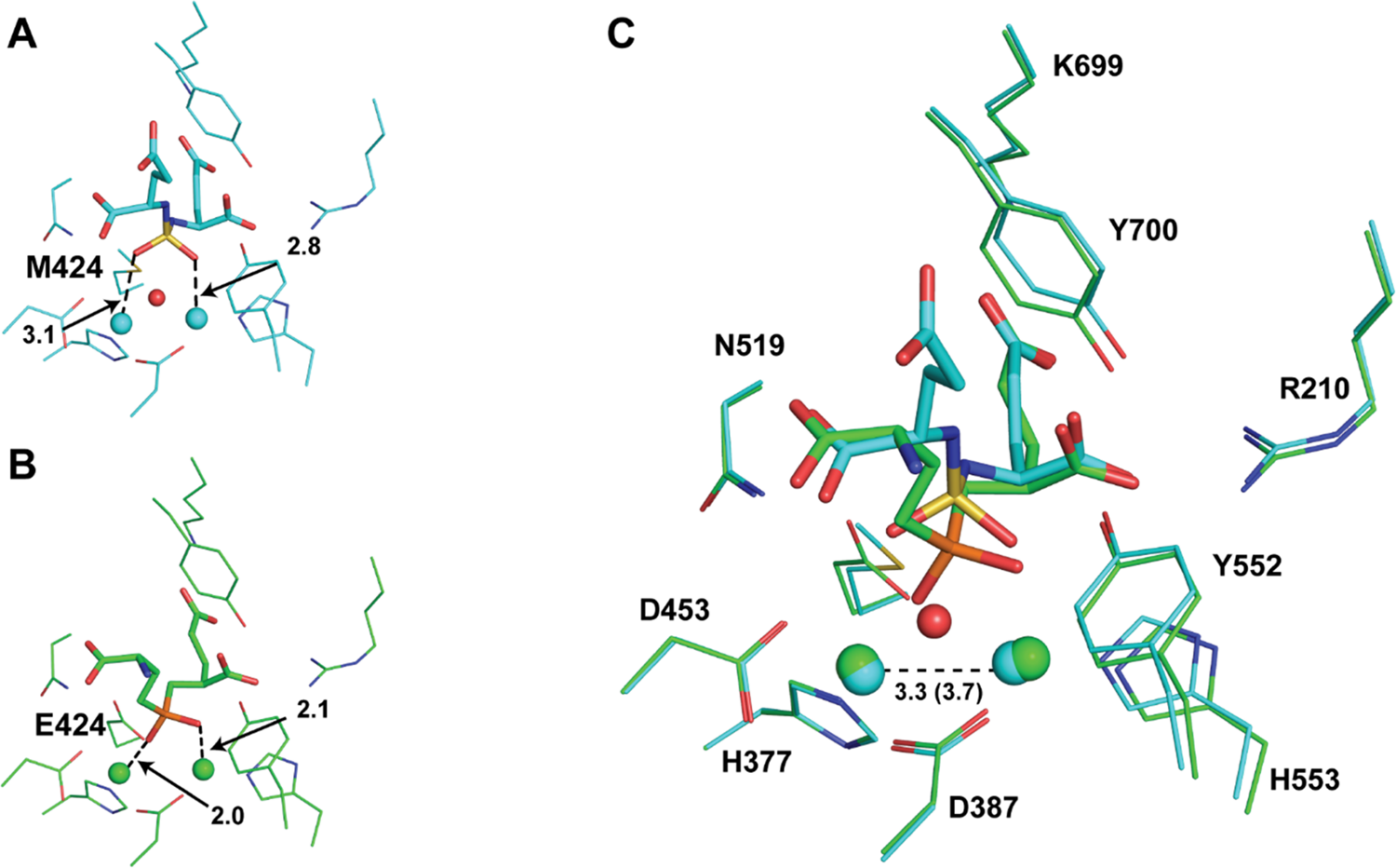
Superposition of X-ray structures of PSMA/**4** and PSMA/**EPE** (PDB code: 3BI0). (A, B) Details of the positioning of **4** (A) and **EPE** (B) in the active site of PSMA. While oxygen atoms of EPE phosphinate form dative bonds with zinc ions (green spheres) at “typical” distances of approximately 2.0 Å, corresponding oxygen atoms of the sulfamide moiety of compound **4** are in the second coordination shell of >2.8 Å. Inhibitors are shown in stick representations with atoms colored red (oxygen), blue (nitrogen), yellow (sulfur), orange (phosphorus), cyan (carbon, compound **4**), and green (carbon, **EPE**). The activated water molecule/OH^–^ anion is shown as a red sphere, while zinc ions are shown as cyan and green spheres for PSMA/**4** and PSMA/**EPE**, respectively. (C) Superposition of the PSMA/**4** and PSMA/**EPE** complexes. Notice differences in the zinc coordination spheres as well as distances between the zinc ions in respective complexes (a number in parentheses for the PSMA/**EPE** complex). The distances are shown in angstroms.

Prior studies have shown that secondary sulfamides maintain the possibility to coordinate the Zn ion *via* the amino group in the deprotonated form, yet such substitutions typically lead to a decrease in their inhibitory potency.^35−37^ Surprisingly, our X-ray structures of PSMA complexes with N^1^-functionalized secondary sulfamides revealed that the sulfamide function does not coordinate the active-site zinc ions but rather serves as a passive linker connecting the P1 and P1′ moieties of the inhibitors. First, sulfamide positioning varies between the two complexes pointing toward the absence of any strong zinc interactions. Second, in both structures, oxygen/nitrogen atoms of the sulfamide function are >2.8 Å away from zinc ions at a distance far exceeding a typical [oxygen/nitrogen···Zn] dative bond of approximately 2.0 Å (Figure 6A). Finally, even with additional interactions with the arginine patch at the S1 site of PSMA mediated by the newly added functions, the potency of bifunctional compounds **3** and **4** is lower compared to the monosubstituted inhibitor **9**, arguing for the loss of sulfamide–zinc bonding. It shall also be noted that the positioning of the terminal amino group of **9** between the active-site zinc ions has undesired consequences for the design and affinity of bisubstituted sulfamides. As the P1 functionality is attached at the terminal amino group (and not via sulfamide oxygens), such substitution leads to steric clashes with active-site zinc ions of the enzyme and will not be tolerated.

The absence of the zinc-binding capacity of bisubstituted sulfamides, which stems from the missing negative charge of the function, is obviously the underlying cause of the low inhibitory potency of compounds **3** and **4**. This low potency is contrasted with the high affinity structurally matching phosphonates/phosphinates, where the negatively charged phosphorus function coordinates the zinc ions faithfully mimicking the tetrahedral reaction intermediate and which are approximately 10^4^–10^5^-fold more potent (Figure 1; ref (18)). The superposition of the PSMA/**4** and PSMA/**EPE** (PDB 3BI0) complexes clearly illustrates differences in coordination of the active-site zinc ions by the two inhibitors with phosphinate oxygen atom position in the first coordination shell on Zn^2+^ ions. Interestingly, the phosphorus-based analogues also displace the activated (catalytic water) hydroxide anion from the active site, while the anion is present in PSMA complexes with secondary sulfamides **3** and **4**, suggesting that the sulfamides will be prone to PSMA-catalyzed hydrolysis that could negatively influence inhibitor stability and its residence time within the active site of the enzyme (Figure 6).

**Figure 7:**
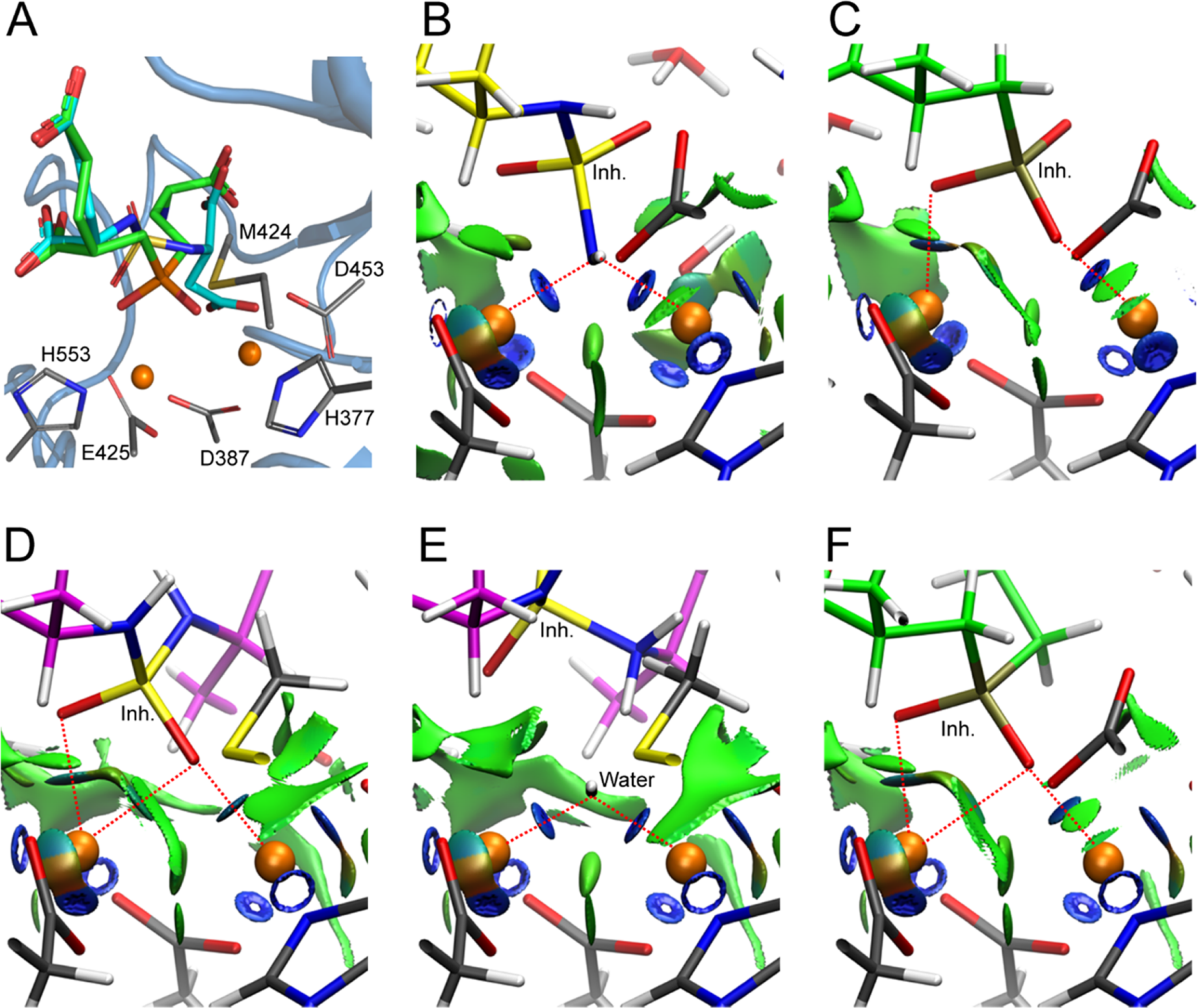
Noncovalent interaction (NCI) analysis of QM-optimized and experimental models. (A) Superposition of the QM-optimized PSMA/**3-direct** (inhibitor carbon atoms cyan) and experimental PSMA/**EPE** (inhibitor carbon atoms green; PDB code 3BI0) complexes. Zinc ions are shown as orange spheres, and PSMA carbon atoms are colored dark gray. (B–F) Noncovalent interaction surface plots for optimized geometries in the dizinc active site of PSMA/inhibitor complexes obtained at the DFT-D3/TPSS/def2-TZVP level with the water-COSMO model. Surfaces are assigned with specific colors to denote the strength and characteristics of the interatomic interactions: green surfaces denote weak van der Waals (vdW) interactions and blue surfaces denote strong attractive interactions. Inhibitors and protein residues are shown in stick representation, and zinc ions are shown as gray spheres. Panels show (B) the PSMA/9-NH-N-complex, (C) PSMA/**2-PMPA** (PDB code 2PVW), (D) PSMA/**3-direct**, (E) PSMA/**3-Water**, and (F) PSMA/**EPE**. The directions of interactions between coordinated ligands or water/hydroxide atoms with zinc ions are highlighted by dashed red lines.

To investigate stability of a hypothetical PSMA/**3** complex, where the sulfamide would adopt a gem-diolate coordination observed in PSMA complexes with phosphorus-based inhibitors,^18,23,38^ we perturbed *in silico* the experimental structure of PSMA/**3** to mimic the geometry of the high-affinity PSMA/**EPE** complex reported previously (PDB code 3BI0; Figure 7). Such a hypothetical QM-optimized PSMA/**3** complex, denoted PSMA/**3-direct**, is shown in Figure 7D, and its superposition with the PSMA/**EPE** complex is shown in Figure 7A. The PSMA/**3-direct** complex is energetically significantly less stable (by approximately 14 kcal/mol) compared to PSMA/**3-Water**, as observed in the X-ray structure. Further, geometry minimization of PSMA/**3-direct** with the added water molecule leads to a barrierless conversion to the PSMA/**3-Water** complex, thus corroborating our experimental findings.

The zinc-binding capacity of various sulfamide configurations reported herein was further examined by a noncovalent interaction (NCI) analysis of QM-optimized models and compared to the NCI data obtained for phosphonates/phosphinates in their crystal structure geometries. The NCI provides a qualitative description of the variability of the electronic structure around the bimetallic active site, comprising selected zinc ions, PSMA residues, inhibitors, and water or OH^–^. The PSMA/**9-NH-1** complex is compared to the structurally matching PSMA/**2-PMPA** complex (PDB code 2PVW; Figure 7B,C), while PSMA complexes with bisubstituted sulfamide **3** in **3-direct** and **3-Water** configurations are compared to the PSMA/**EPE** complex (PDB code 3BI0; Figure 7D–F). The NCI results indicate strong stabilizing interactions between zinc atoms and the coordinated water/OH^–^ moiety and phosphonates/phosphinate inhibitors (shown as blue surfaces in Figure 7), while sulfamide functions are accommodated in the dizinc active site mostly by weaker interactions (cyan surfaces) complemented by weak van der Waals interactions (green surfaces). NCI surface plots for the PSMA/**3-OH** and PSMA/**4** complexes are shown in Figure S2.

## Conclusions

Structural, biochemical, and computational analyses reported here provide experimental evidence that while the sulfamide function is an excellent building block for medicinal bioactive compounds, due to their suboptimal metal–liganding properties, uncharged bisubstituted sulfamides may be less advantageously used in a setting where they are designed to directly engage metal ions of targeted metalloproteins. In our study case involving PSMA, they are, in terms of their binding affinity, clearly outperformed by structurally matching negatively charged phosphinates/phosphonates. At the same time, further studies are warranted to provide a better understanding of the impact of incorporation of electron-withdrawing groups into such metalloprotein inhibitors^34^ or exploiting related sulfur-containing function groups, such as sulfondiimines or sulfoximines.^39^

## Materials and Methods

### Inhibitor Synthesis

All reagents and solvents were purchased from commercial suppliers and used without purification, unless otherwise noted. Column chromatography was carried out using an ISCO CombiFlash Companion with prepacked GOLD grade silica or C18 columns. ^1^H and ^13^C NMR spectra were recorded on a Varian 300 MHz spectrometer or on an Agilent 400 MHz spectrometer (analytical data shown in Figure S2). All spectra were determined in the solvents indicated, ^1^H NMR chemical shifts are relative to those of CDCl_3_ (*d* = 7.26 ppm) or D_2_O (*d* = 4.79 ppm). ^13^C NMR chemical shifts are relative to CDCl_3_ (*d* = 77.23 ppm). High-resolution mass spectra were obtained on an Agilent 7200 Q-TOF mass spectrometer. All compounds reported in the manuscript are >95% pure as determined by NMR analysis (Figure S3).

#### (*S*)-Dimethyl 2-(chlorosulfonylamino)butanedioate (**5**)

To a stirred solution of sulfuryl chloride (11.40 g, 84.40 mmol) in acetonitrile (13 mL) was added (*S*)-dimethyl aspartate hydrochloride (5.00 g, 25.32 mmol). Upon complete addition, the reaction mixture was stirred for 24 h at reflux. Volatile materials were evaporated in vacuum to a viscous oil, which was extracted by stirring with diethyl ether (50 mL) for an hour. The resulting precipitate was filtered through a Celite pad, the Celite pad was washed with additional diethyl ether (20 mL) and the combined solutions were concentrated in vacuo to yield crude sulfamoyl chloride **5** (5.08 g, 77%) as a colorless viscous oil, which was used in the subsequent step without further purification. ^1^H NMR (300 MHz, CDCl_3_) δ = 7.16 (d, *J* = 7.8 Hz, 1H), 4.52 (dt, *J* = 8.5, 4.3 Hz, 1H), 3.86 (s, 3H), 3.71 (s, 3H), 3.12, 2.93 (m, 2H). ^13^C NMR (75 MHz, CDCl_3_) δ = 172.83, 170.41, 54.72, 53.47, 52.25, 37.66.

#### (*S*)-Dimethyl 2-(chlorosulfonylamino)pentanedioate (**6**)

Compound **6** was prepared analogously as compound **5**. From (*S*)-dimethyl glutamate hydrochloride (5.00 g, 23.65 mmol), sulfuryl chloride (10.64 g, 78.80 mmol), and acetonitrile (12 mL) was prepared sulfamoyl chloride **6** (5.19 g, 80%) as a colorless viscous oil, which was used in subsequent steps without further purification. ^1^H NMR (300 MHz, CDCl_3_) δ = 6.97 (d, *J* = 7.0 Hz, 1H), 4.35 (td, *J* = 8.2, 4.7 Hz, 1H), 3.85 (s, 3H), 3.69 (s, 3H), 2.60–2.40 (m, 2H), 2.35–2.40 (m, 1H), 2.14–2.02 (m, 1H). ^13^C NMR (75 MHz, CDCl_3_) δ = 172.96, 170.55, 56.65, 53.52, 52.11, 29.37, 27.46.

#### (*S*)-2-(Sulfoamino)butanedioic Acid Trisodium Salt (**1**)

To a solution of **5** (0.78 g, 3.00 mmol) in THF (12 mL) was added a methanolic solution of LiOH·H_2_O (1 M, 13.5 mL). The resulting solution was stirred at room temperature until completion. Then, the reaction mixture was acidified (pH = 3) with 4 M HCl and the solvent was evaporated in vacuum. The crude residue was purified via chromatography on a C18 column (5–97% methanol in water) to afford a glassy solid (0.52 g, 2.43 mmol). This noncrystalline material was dissolved in a solution of NaHCO_3_ (0.61 g, 7.29 mmol, 3 equiv) in water (10 mL), evaporated, and crystallized from methanol to afford **1** (0.60 g, 71%) as colorless crystals, mp >250 °C. ^1^H NMR (400 MHz, D_2_O) δ = 3.88 (dd, *J* = 8.6, 3.8 Hz, 1H), 2.79 (dd, *J* = 17.5, 3.8 Hz, 1H), 2.66 (dd, *J* = 17.5, 8.6 Hz, 1H). ^13^C NMR (100 MHz, D_2_O) δ = 177.41, 174.14, 52.05, 36.38. HRMS calcd for C_4_H_6_NO_7_S- 211.9870 [M – H]^−^; found 211.9870.

#### (*S*)-2-(Sulfoamino)pentanedioic Acid (**2**)

To a solution of **6** (1.00 g, 3.66 mmol) in THF (15 mL) was added a methanolic solution of LiOH·H_2_O (1 M, 16.5 mL). The resulting solution was stirred at room temperature until completion. Then, the reaction mixture was acidified (pH = 3) with 4 M HCl and the solvent was evaporated in vacuum. The crude residue was purified via chromatography on a C18 column (5–97% methanol in water) to afford **2** (0.73 g, 79%) as colorless crystals, mp 157–159 °C. ^1^H NMR (400 MHz, D_2_O) δ = 4.36 (dd, *J* = 9.2, 5.0 Hz, 1H), 2.62, 2.46 (m, 1H), 2.45, 2.33 (m, 2H), 2.20, 2.11 (m, 1H). ^13^C NMR (100 MHz, D_2_O) δ = 181.78, 176.45, 55.84, 29.10, 24.30. HRMS calcd for C_5_H_9_NO_7_S^–^ [M – H]^−^ 226.0027 [M – H]^−^; found 226.0025.

#### (*S*)-Dimethyl 2-((*N*-((*S*)-1,4-dimethoxy-1,4-dioxobutan-2-yl)sulfamoyl)amino)pentanedioate (**7**)

To a stirred solution of (*S*)-dimethyl glutamate hydrochloride (0.93 g, 4.39 mmol) and **5** (1.04 g, 4.00 mmol) in dichloromethane (30 mL) cooled in an ice bath was added triethylamine (0.75 mL, 5.38 mmol). The mixture was stirred for 30 min at room temperature and then extracted with 1 M HCl (2 × 15 mL) and brine (15 mL). The organic phase was dried over Na_2_SO_4_ and evaporated. The resulting crude residue was purified via silica gel chromatography (hexane/EtOAc 7:3) to yield **7** as a clear thick oil (1.15 g, 72%). ^1^H NMR (300 MHz, CDCl_3_) δ = 5.69 (d, *J* = 8.8 Hz, 1H), 5.48 (d, *J* = 9.1 Hz, 1H), 4.27 (dt, *J* = 8.5, 4.4 Hz, 1H), 4.12 (td, *J* = 8.8, 5.0 Hz, 1H), 3.76 (s, 6H), 3.69 (s, 3H), 3.67 (s, 3H), 3.05–2.88 (m, 2H), 2.57–2.38 (m, 2H), 2.27–2.15 (m, 1H), 2.00–1.88 (m, 1H). ^13^C NMR (75 MHz, CDCl_3_) δ = 172.37, 172.22, 170.60, 170.30, 55.64, 53.52, 53.41, 52.73, 52.36, 52.12, 37.30, 29.90, 27.67.

#### (2*S*,2′*S*)-Tetramethyl 2,2′-(Sulfonylbis(azanediyl))dipentanedioate (**8**)

Compound **8** was prepared analogously as compound **7**. From (*S*)-dimethyl glutamate hydrochloride (1.00 g, 4.73 mmol), **6** (1.18 g, 4.30 mmol) in dichloromethane (30 mL) and triethylamine (0.75 mL, 5.38 mmol) was prepared **8** as a clear thick oil (1.44 g, 81%). ^1^H NMR (300 MHz, CDCl_3_) δ = 5.35 (d, *J* = 8.8 Hz, 2H), 4.07 (td, *J* = 8.4, 4.8 Hz, 2H), 3.75 (s, 3H), 3.66 (s, 3H), 2.55–2.36 (m, 4H), 2.23–2.13 (m, 2H), 1.99–1.87 (m, 2H). ^13^C NMR (75 MHz, CDCl_3_) δ = 172.36, 170.10, 55.34, 53.41, 52.14, 29.77, 27.84.

#### (*S*)-2-((*N*-((*S*)-1,2-Dicarboxyethyl)sulfamoyl)amino)pentanedioic Acid Tetrasodium Salt (**3**)

To a solution of **7** (0.50 g, 1.26 mmol) in THF (10 mL) was added a methanolic solution of LiOH (1 M, 10.0 mL). The resulting solution was stirred at room temperature until completion. The reaction mixture was acidified (pH = 3) with 4 M HCl and the solvent was evaporated in vacuum. The crude residue was purified via chromatography on a C18 column (5–97% methanol in water) to afford a glassy solid (0.35 g, 1.02 mmol). This noncrystalline material was dissolved in a solution of NaHCO_3_ (0.34 g, 4.07 mmol, 4 equiv) in water (7 mL), evaporated, and crystallized from methanol to afford **3** (0.35 g, 65%) as colorless crystals, mp >250 °C ^1^H NMR (400 MHz, D_2_O) δ = 3.86 (dd, *J* = 8.2, 4.4 Hz 1H), 3.62–3.55 (m, 1H), 2.51 (dd, *J* = 15.5, 4.7 Hz 1H), 2.36 (dd, *J* = 15.5, 8.5 Hz, 1H), 2.15–2.05 (m, 2H), 1.88–1.65 (m, 2H). ^13^C NMR (100 MHz, D_2_O) δ = 182.20, 179.26, 178.88, 178.54, 58.05, 56.11, 40.71, 33.60, 29.23. HRMS calcd for C_9_H_12_N_2_O_10_S- 341.0296 [M – H]^−^; found 341.0299.

#### (2*S*,2′*S*)-2,2′-(Sulfonylbis(azanediyl))dipentanedioic Acid (**4**)

To a solution of **5** (0.55 g, 1.33 mmol) in THF (10 mL) was added a methanolic solution of LiOH (1 M, 11.0 mL). The resulting solution was stirred at room temperature until completion. The reaction mixture was acidified (pH = 3) with 4 M HCl and the solvent was evaporated in vacuum. The crude residue was purified via chromatography on a C18 column (5–97% methanol in water) to afford **4** (0.33 g, 72%) as colorless crystals, mp 179–181 °C. ^1^H NMR (400 MHz, D_2_O) δ = 3.72–3.55 (m, 2H), 2.29–2.13 (m, 4H), 2.01–1.73 (m, 4H). ^13^C NMR (100 MHz, D_2_O) δ = 182.08, 179.20, 58.07, 33.73, 29.28. HRMS calcd for C_10_H_14_N_2_O_10_S- 355.0453 [M – H]^−^; found 355.0457.

### Site-Directed Mutagenesis

The PSMA(E424M) mutant was constructed by a Quick-change site-directed mutagenesis protocol using the plasmid encoding N-terminally tagged wild-type PSMA^40^ as a template, together with a pair of complementary mutagenic primers Met(E424M)_F: 5′- GCAAGCTGGGATGCAATGGAATTTGGTCTTCTTG-3′ and Met(E424M)_R: 5′-CAAGAAGACCAAATTCCATTGCATCCCAGCTTGC-3′. The identity of the resulting expression construct was verified by Sanger sequencing.

### Expression and Purification of PSMA Variants

Expression and purification of the extracellular part of human PSMA variants (wild-type PSMA and the PSMA(E424M) mutant; amino acids 44–750) were performed essentially as described previously.^40^ Briefly, protein fusions were overexpressed in Schneider’s S2 cells and concentrated and dialyzed using tangential flow filtration (Millipore, Mosheim, France). The purification protocol included affinity chromatography on Streptactin Sepharose (IBA, Göttingen, Germany), followed by size-exclusion chromatography on a Superdex 200 column (GE Healthcare Bio-Sciences, Uppsala, Sweden) with the mobile phase, comprising 20 mM Tris–HCl and 150 mM NaCl, pH 8.0. Purified PSMA variants were concentrated to 10 mg/mL and kept at −80 °C until further use.

### Determination of Inhibition Constants

Inhibition constants of studied sulfamides were determined using the radioenzymatic assay with ^3^H-NAAG (radiolabeled at the terminal glutamate) as a substrate essentially as described previously.^41^ Briefly, PSMA (30 ng/mL) was preincubated in the presence of increasing concentrations of inhibitors in 20 mM Tris–HCl, 150 mM NaCl, and 0.001% C_12_E_8_, pH 8.0, for 15 min at 37 °C in the total volume of 80 μL in a polypropylene 96-well plate. Reactions were initiated by addition of 40 μL of 0.31 μM NAAG (Sigma) and 15 nM ^3^H-NAAG (50 Ci/mmol, PerkinElmer) mixture and terminated after 20 min by the addition of 120 μL of 200 mM potassium phosphate, 50 mM EDTA, 2 mM β-mercaptoethanol, pH 7.4. The released glutamate was separated from the reaction mixture by ion-exchange chromatography (AG 1-X8 formate resin, BioRad) and quantified by liquid scintillation. Duplicate reactions were carried out for each experimental point. The data were fitted using GraphPad Prism software (GraphPad Software, San Diego, CA, USA), and IC_50_ values were calculated from the inhibition curves of two independent experiments using a nonlinear analysis protocol.

### Crystallization and Data Collection

Crystallization experiments were carried out using established protocols.^38^ Diffracting crystals of PSMA/sulfamide complexes were obtained by first mixing the PSMA stock solution (10 mg/mL) with an inhibitor (20 mM in water) at a 9:1 (v/v) ratio and then combining the PSMA/sulfamide solution with the same volume of the reservoir solution [33% pentaerythritol propoxylate (Sigma), 1.5% poly(ethylene glycol) 3350 (Sigma), and 100 mM Tris–HCl, pH 8.0]. Crystals were grown in the hanging-drop vapor-diffusion setup at 293 K and diffraction intensities were collected from a single crystal at 100 K using synchrotron radiation at the MX14.2 beamline BESSYII, Helmholtz-Zentrum Berlin, Germany (PSMA/**9** complex), and P13 (MX1) beamline PETRA III, EMBL C/O DESY, Hamburg, Germany (PSMA/**3** and PSMA/**4**). Diffraction data were indexed, integrated, and scaled using MOSFLM^42^ and XDS^43^ programs in the XDSAPP interface.^44^

### Structure Determination and Refinement

The structures of all complexes were determined by difference Fourier methods with the ligand-free PSMA structure (PDB code 2OOT) used as a starting model.^45^ The PRODRG server was used to generate the restrain libraries and the coordinate files for the inhibitors.^46^ Calculations were performed with the program Refmac 5.5. and the structural refinement was interspersed by manual corrections to the models using Coot 0.6.^47,48^ The quality of the final models was elucidated using MolProbity.^49^ Data collection and refinement statistics are summarized in Table 1. Atomic coordinates of the present structures together with the experimental structure factor amplitudes of PSMA/**9**, PSMA/**3**, and PSMA/**4** complexes were validated and deposited at the Protein Data Bank^50^ under accession numbers 4W9Y, 6SKH, and 6SGP, respectively.

### QM Cluster Model Calculations

All structural models used in QM cluster calculations were based on the investigated X-ray structures. The QM model consists of relevant atoms/residues of the active site related to the chemical process under consideration and their neighbors.

PSMA complex with monosubstituted glutamyl sulfamide (the PSMA/**9** complex, including 327 atoms): two zinc ions, inhibitor atoms, and all atoms in Asn257, Glu424, Glu425, Phe426, Leu428, Asn519, Lys699, and Tyr700 residues. The fragments of residues Phe209, Arg210, Leu258, His377, Trp381, Asp387, Gly427, Asn451, Asp453, Gly518, Arg534, Tyr552, and His553 are capped by the methyl group and atoms of a hydrogen-bonded water network.

PSMA(E424M) mutant complex with bisubstituted sulfamide (**3**, including 367 atoms and **4**, including 376 atoms): two zinc ions, inhibitor atoms, catalytic water/OH^–^ atoms, all atoms in Gly256, Met424, Glu425, Ser456, Glu457, Lys699, and Tyr700 residues. The fragments of residues Phe209, Arg210, Leu258, His377, Trp381, Asp387, Leu428, Asn451, Asp453, Arg463, Gly518, Asn519, Arg534, Arg536, Tyr552, His553, and Tyr559 are capped by the methyl group and atoms of a hydrogen-bonded water network.

The protonation state of histidine residues in the QM cluster model was assigned as “HID” for both His377 and His553 residues based on the hydrogen bond network and solvent accessibility around the residue of interest. Geometry optimizations were carried out at the DFT-D3/TPSS level, using def2-SVP basis set for all atoms, with resolution-of-identity (RI-J) approximation in the def2-SVP auxiliary basis set in the conductor-like screening solvation model in water (COSMO solvation model with ε = 78.4).^51−53^ The single-point energies were calculated using the RI-TPSS dispersion corrected density functional (DFT-D3) with the def2-TZVP basis set and def2-TZVP auxiliary basis set for all atoms in water-COSMO as implemented in Turbomole 6.5 program (http://www.turbomole.com).^54^ To assess the effects of the dielectric constant value used, we also performed the single-point calculation using ε values of 4, 8, 20, and 40. All NCI calculations were performed with the NCIPLOT program^55,56^ on top of optimized QM cluster models using the promolecular density approximation analyzing interactions between zinc atoms and surrounding residues/ligands within 3.5 Å. The density isovalue of 0.3 e was used for the visualization in VMD. Classical MD simulations for PSMA/3-Water (PDB 6sgp) and PSMA/4-Water (PDB 6skh) complexes with the CHARMM36 force field were performed in Gromacs as three independent 100 ns MD runs for each system. To maintain the active site structure of PSMA close to the X-ray structure, distance restraints have been applied to the zinc–zinc distance and zinc–O/N-bounded residue distances (i.e., His377, Asp387, Glu425, His553, and Asp453 residues). Position restraint was also applied to oxygen atoms of the zinc-coordinated water in the PSMA models. The +2 charge has been assigned for each Zn atom located in the PSMA active site during MD simulation. Root-mean-square fluctuations (RMSF) of ligand coordinates were calculated from combined 300 ns trajectories by using VMD.

### Analysis of Zn–Sulfamide Complexes in the PDB

Potential Zn–sulfamide complexes were identified in a local mirror of the PDB containing over 202 000 structures as of 2023/06/22. Filtering structures containing S, O, N, and Zn atoms resulted in 4529 candidate structures further processed using a simple RDKit python script searching for the sulfamide [S](O)(O)(N)N substructure. The search identified 60 structures with a zinc atom within 6 Å of the sulfamide. Only three structures contained binuclear zinc centers, our previously solved glutamyl sulfamide complex (4W9Y) and the two structures reported here.

## Data Availability

Atomic coordinates and corresponding structure factors for PSMA/**9**, PSMA/**3**, and PSMA/**4** complexes have been deposited at the Protein Data Bank under accession numbers 4W9Y, 6SKH, and 6SGP, respectively. Geometry coordinates of the optimized structures and relevant Pymol session files as discussed in the main text are provided in a separate archive available at https://zenodo.org/record/10411420. Software used in this manuscript is a third-party software and packages and versions are described in detail in the Materials and Methods section.

## Supporting Information Available

Figure S1 showing noncovalent interaction surface plots of PSMA/inhibitor complexes, Figure S2 contains analytical data of the synthesized compounds, and the source data for Figures 3, 5, 7, and S1, including equilibrium geometries of all studied systems (QM cluster models) are available at https://zenodo.org/record/10411420 (PDF)

## Abbreviations

ZBG: zinc-binding group
CA IX: carbonic anhydrase IX
CPA: carboxypeptidase A
PSMA: prostate-specific membrane antigen
QM: quantum mechanics
