# Supplementary material for: Structural, Biochemical, and Computational Characterization of Sulfamides as Bimetallic Peptidase Inhibitors
Source: J Chem Inf Model. 2024 Jan 15;64(3):1030–42. doi: 10.1021/acs.jcim.3c01542 (PMC10865363; doi:10.1021/acs.jcim.3c01542)
Supplement: Supplementary file 1 — ci3c01542_si_001.pdf [file ci3c01542_si_001.pdf]

Supplementary Materials

Structural, biochemical, and computational characterization of sulfonamides as bimetallic peptidase inhibitors

Zora Novakova[1$], Zahra Aliakbar Tehrani[1$], Radek Jurok[2], Lucia Motlova[1], Zsofia Kutil[1], Jiri Pavlicek[1], Shivam Shukla[1], Cindy J. Choy[3], Barbora Havlinova[1], Petra Baranova[1], Clifford E. Berkman[3], Martin Kuchar[2], Jiri Cerny[1], and Cyril Barinka[1]*

[1]Institute of Biotechnology of the Czech Academy of Sciences, BIOCEV, Prumyslova 595, 252 50 Vestec, Czech Republic

[2]Forensic laboratory of biologically active substances, University of Chemistry and Technology Prague, Technická 3, 166 28 Prague 6, Czech Republic

[3]Department of Chemistry, Washington State University, Pullman, USA

[$]equal contribution

*Address correspondence to: Cyril Barinka, Laboratory of Structural Biology, Institute of Biotechnology of the Czech Academy of Sciences, BIOCEV, Prumyslova 595, 252 50 Vestec, Czech Republic; phone: +420-325-873-777; e-mail: cyril.barinka@ibt.cas.cz

Supporting Information Contents

Figures S1, S2, and S3

Tables S1, S2

Geometry coordinates of the optimized structures and relevant Pymol session files as discussed in the main text are provided in a separate archive available at https://zenodo.org/record/8272552.

RMSF-vmd: PSMA-3 (PDB 6skh)

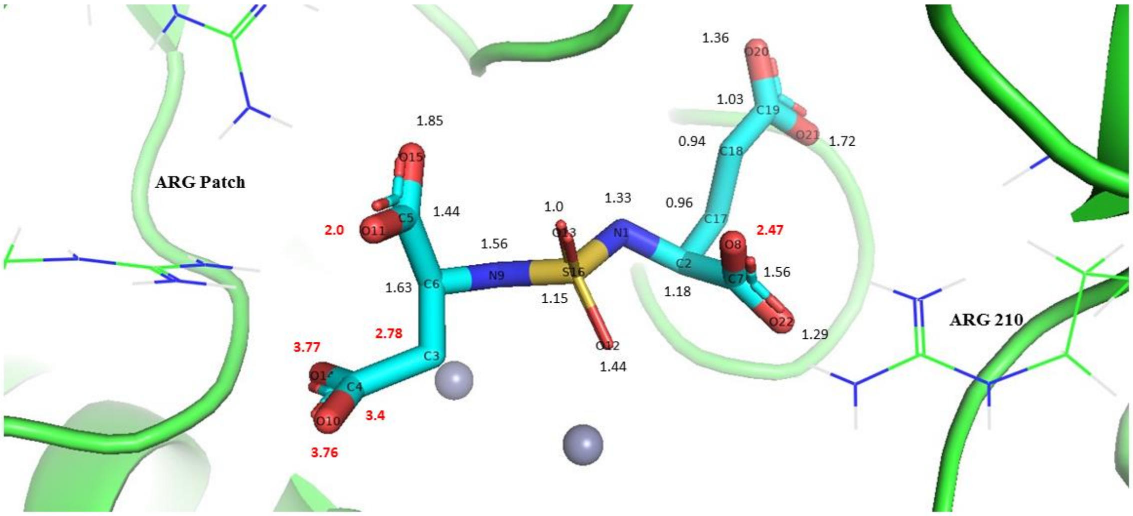

RMSF-vmd: PSMA-4 (PDB 6sgp)

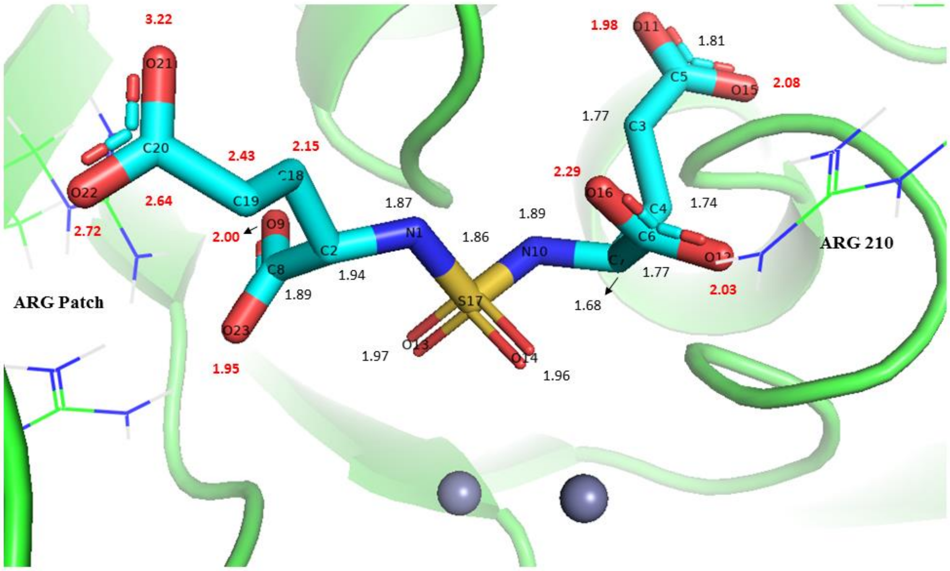

Fig. S1. RMSF values for PSMA-**3** and PSMA-**4** ligand heavy atoms calculated using VMD from three independent 100 ns MD simulations for each ligand starting from the corresponding crystal structure.

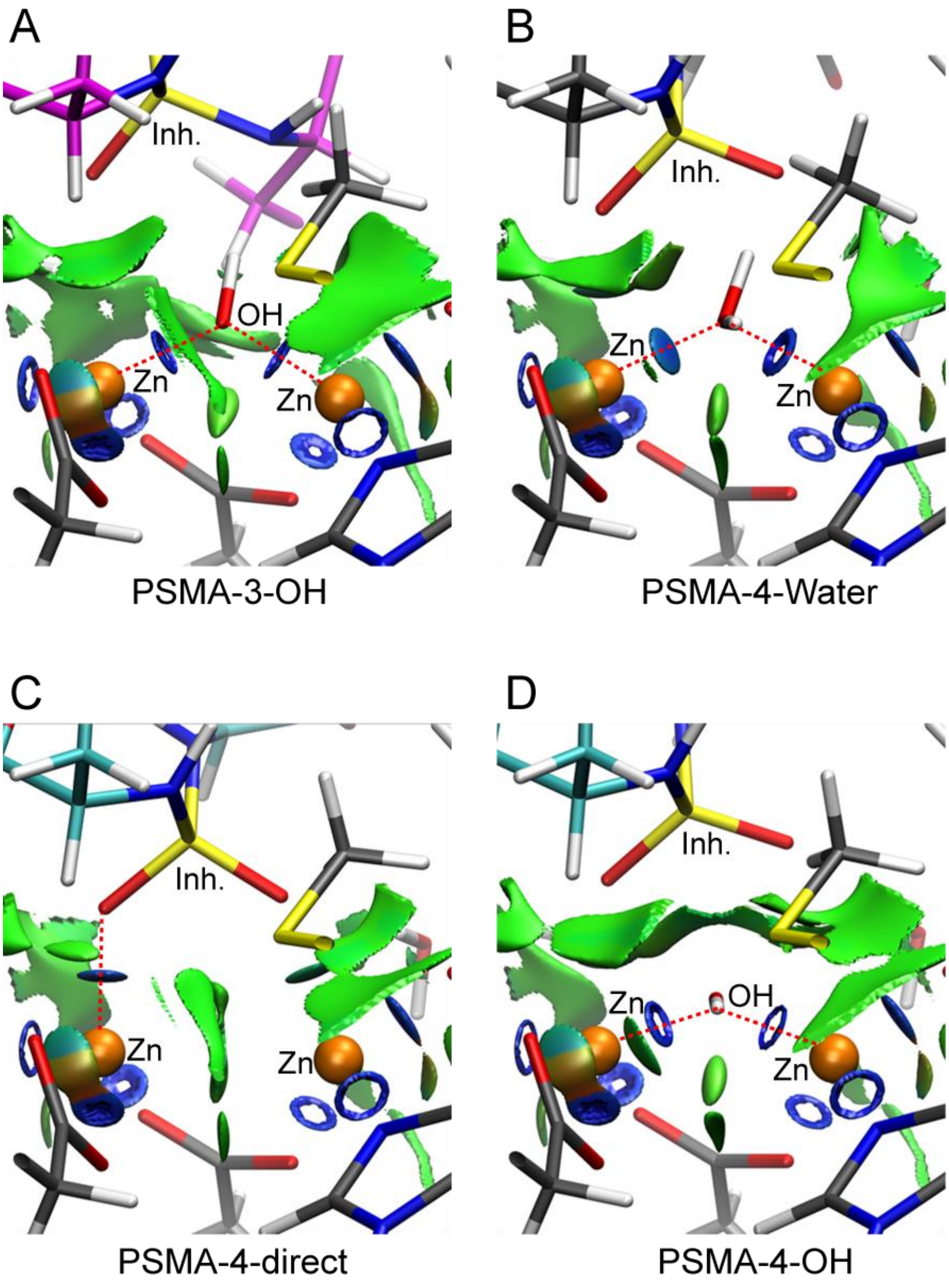

Fig. S2. Non-covalent interaction surface plots for optimized geometries in the di-zinc active site of PSMA/inhibitor complexes obtained at the DFT-D3/TPSS/def2-TZVP level with the water-COSMO model. Surfaces are assigned with specific colors to denote the strength and characteristic of the interatomic

interactions: green surfaces denote weak van der Waals (vdW) interactions and blue surfaces strong attractive interactions. Inhibitors (Inh.) and protein residues are shown in stick representation and zinc ions are shown as orange spheres. Panels show **A)** the PSMA/**3-OH** complex, **B)** PSMA/**4-Water** complex, **C)** PSMA/**4-direct** complex, and **D)** PSMA/**4-OH** complex. The directions of interactions between coordinated ligand or water/hydroxide atoms with zinc ions are highlighted by dashed red lines.

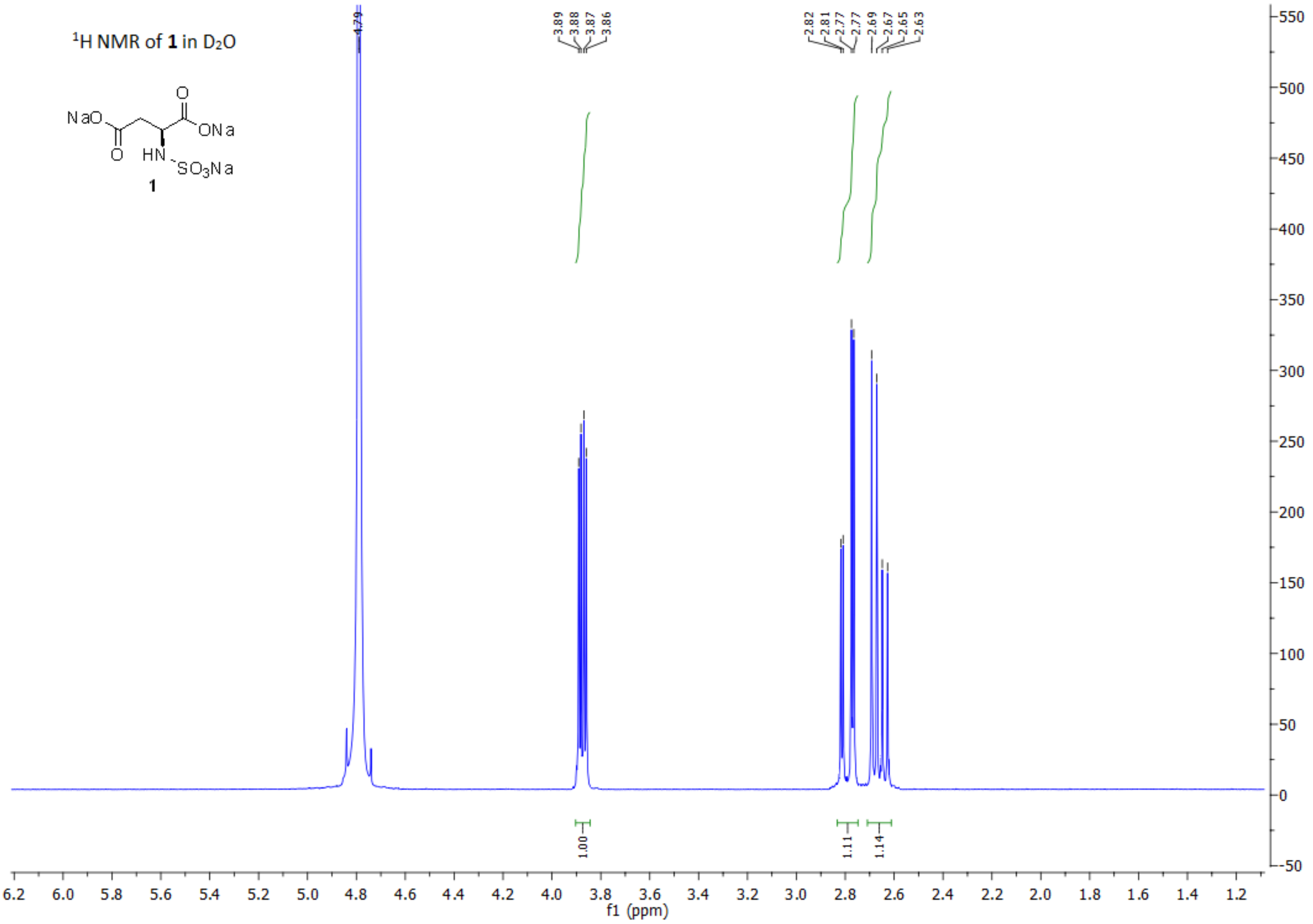

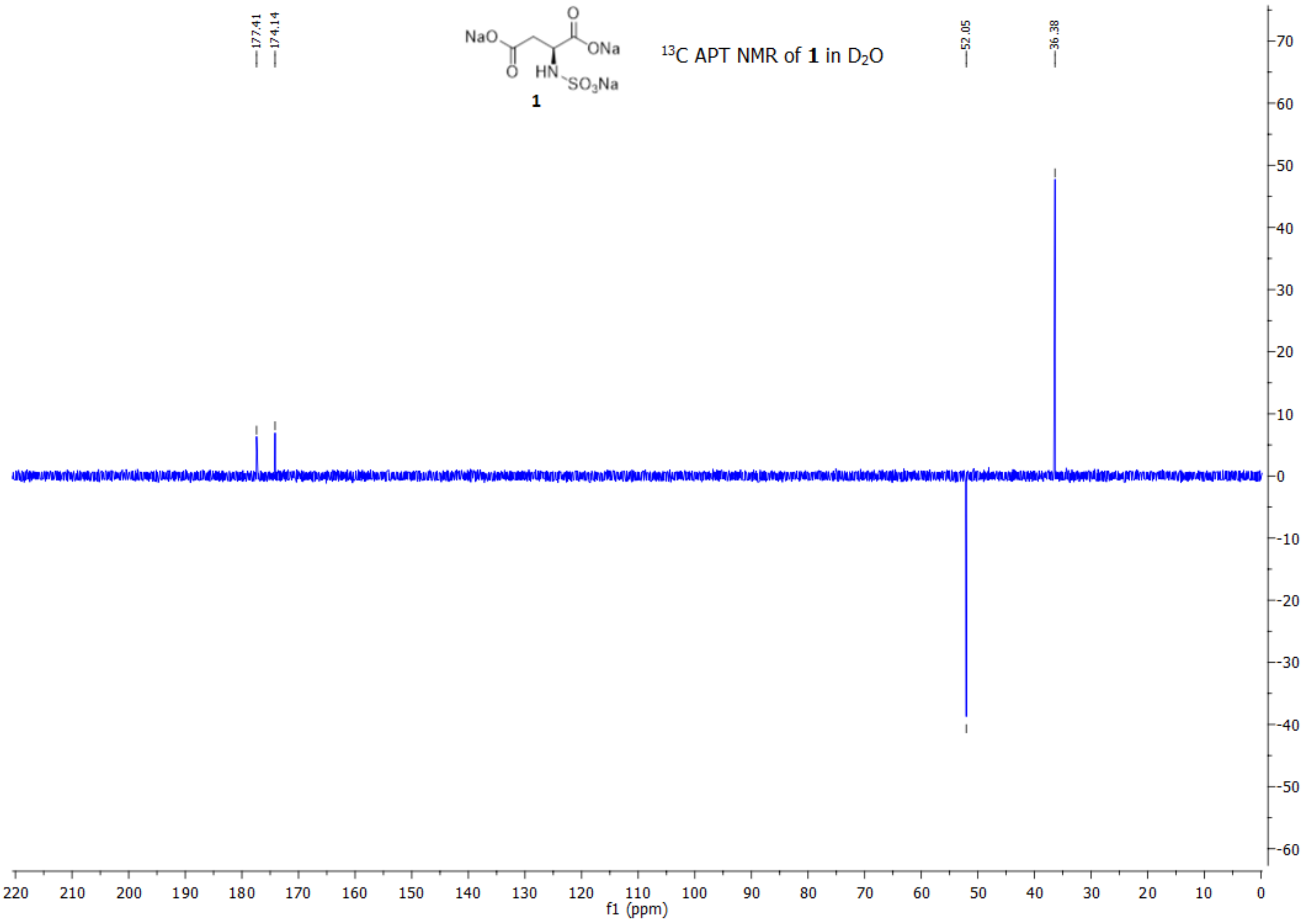
177.41
174.14
NaO
O
O
ONa
HN
SO3Na
1
13C APT NMR of 1 in D2O
52.05
36.38
f1 (ppm)

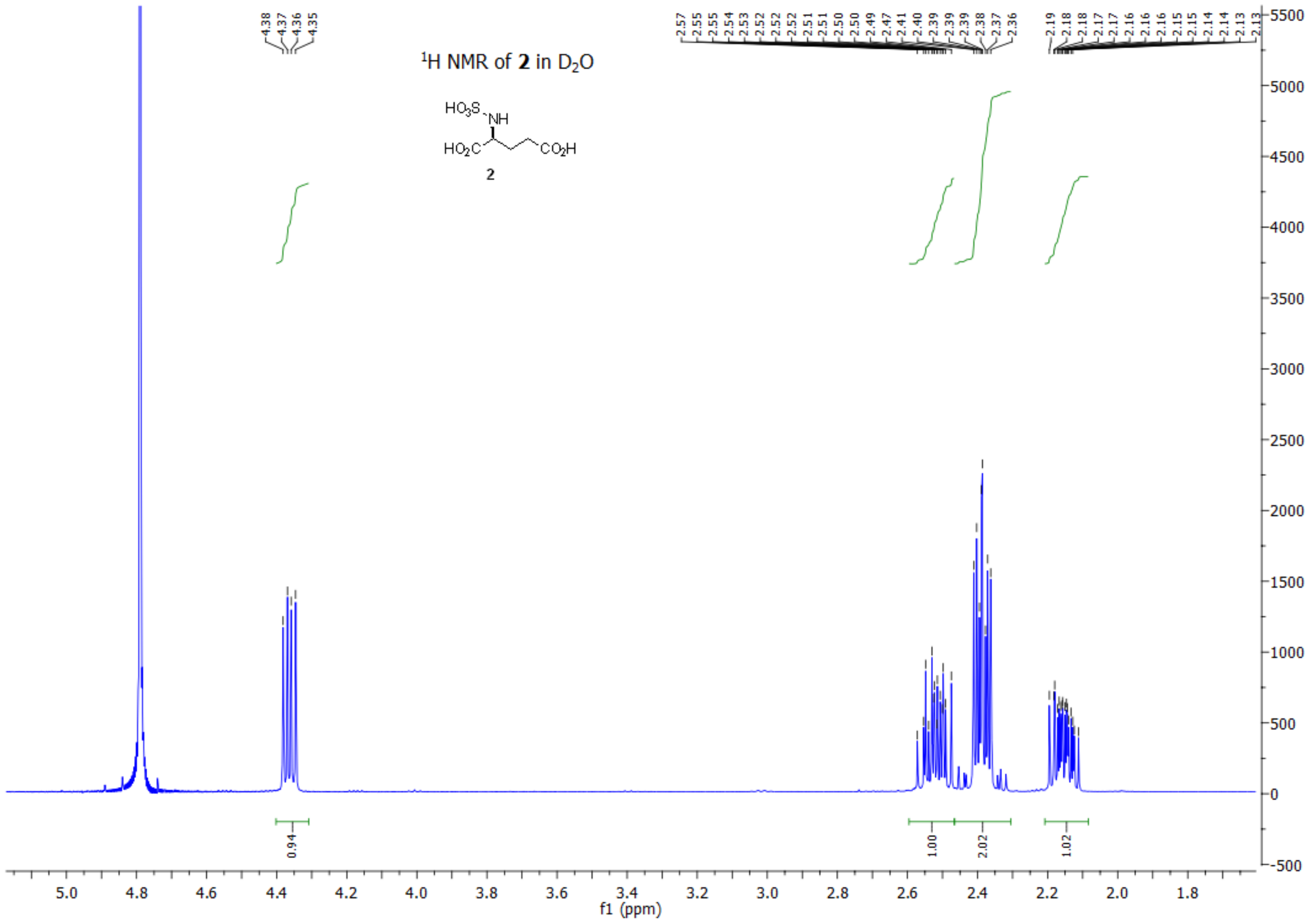

^{1}H NMR of 2 in D2O
HO3S
NH
HO2C
CO2H
2
f1 (ppm)

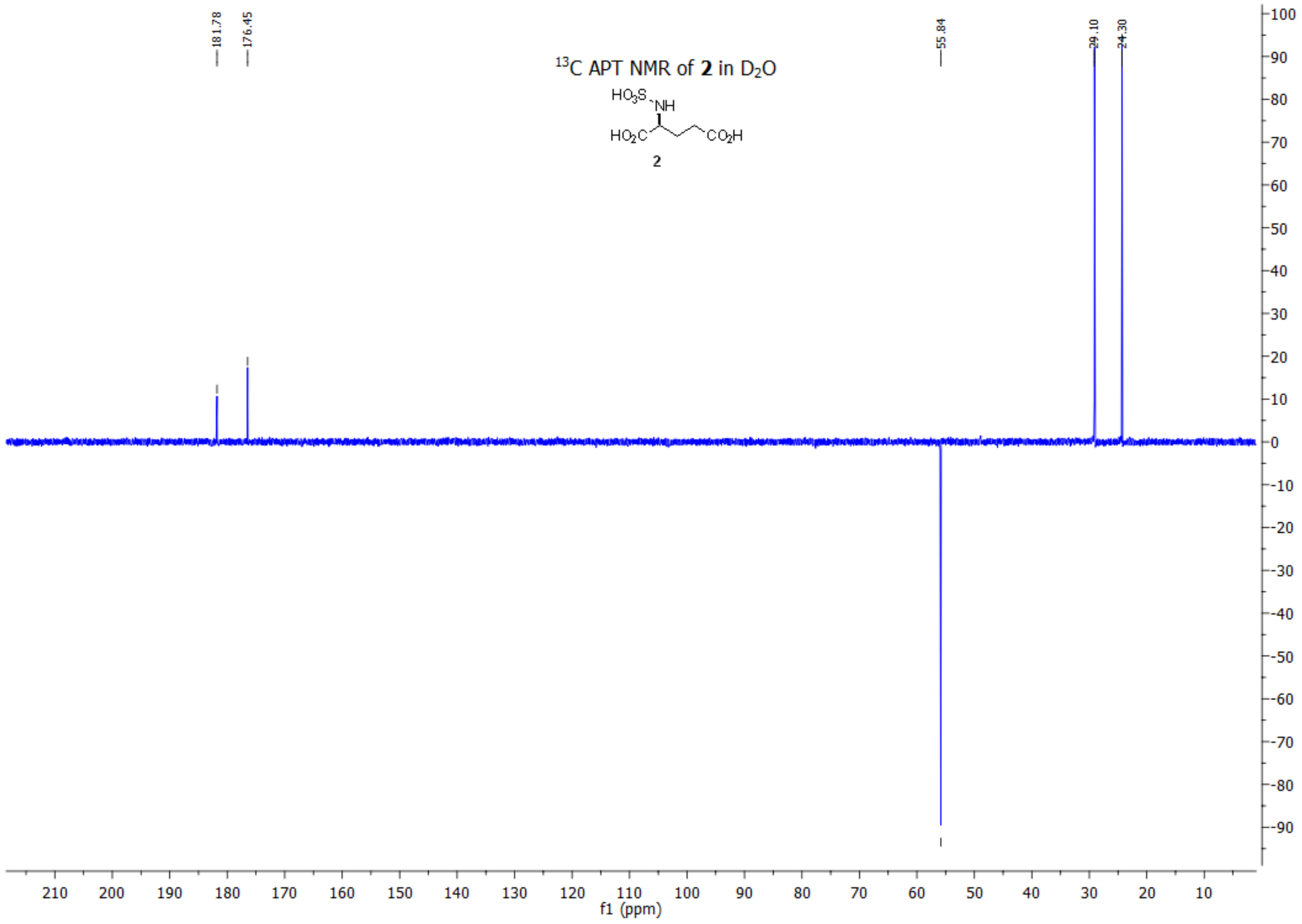
13C APT NMR of 2 in D2O
HO3S
NH
HO2C
CO2H
2
181.78
176.45
55.84
29.10
24.30
f1 (ppm)

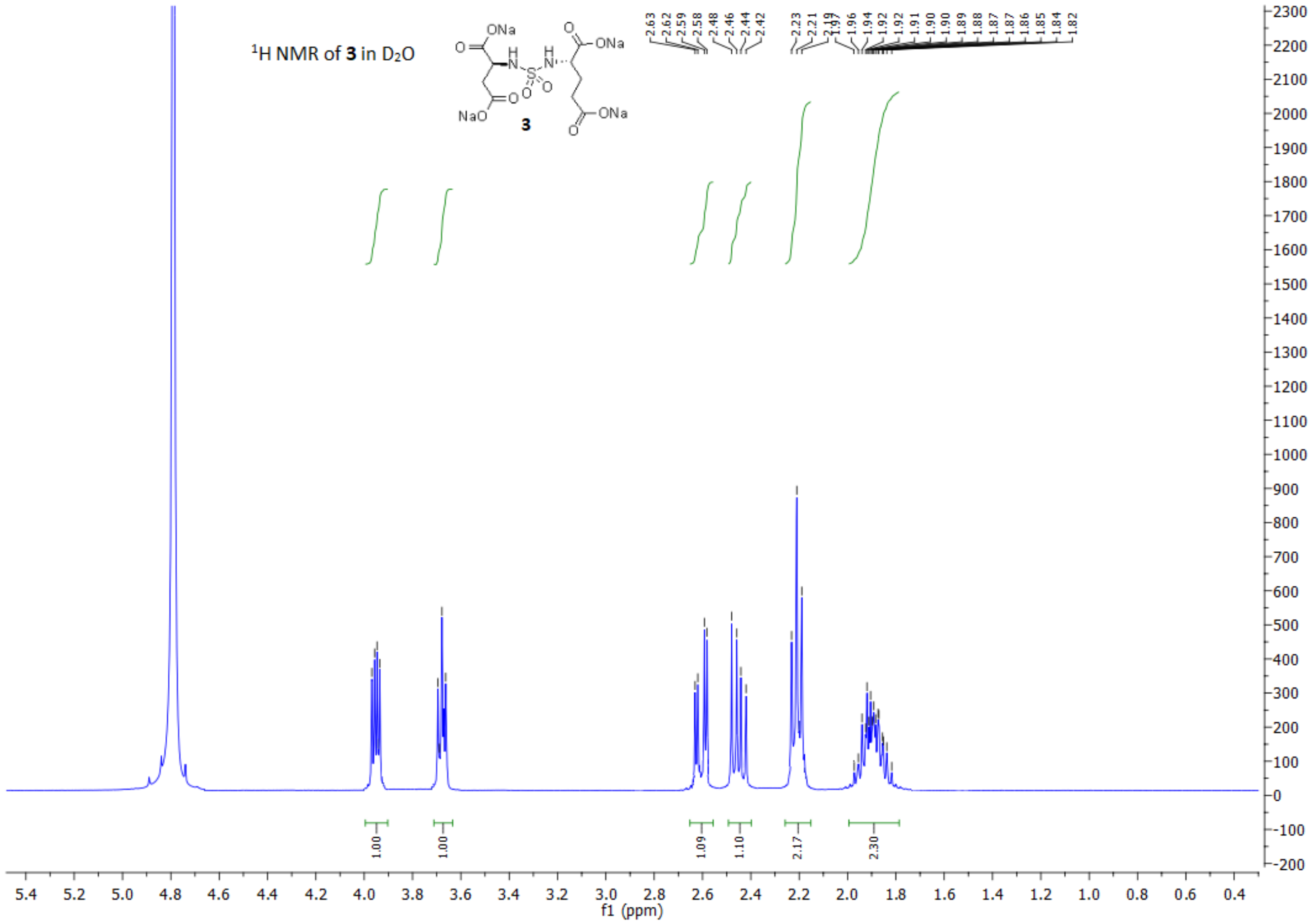

^{1}H NMR of **3** in D_2O
ONa
NaO
ONa
ONa
3
2.63
2.62
2.59
2.58
2.48
2.46
2.44
2.42
2.23
2.21
2.19
1.97
1.96
1.94
1.92
1.92
1.91
1.90
1.90
1.89
1.88
1.87
1.87
1.86
1.85
1.84
1.82
1.00
1.00
1.09
1.10
2.17
2.30
f1 (ppm)

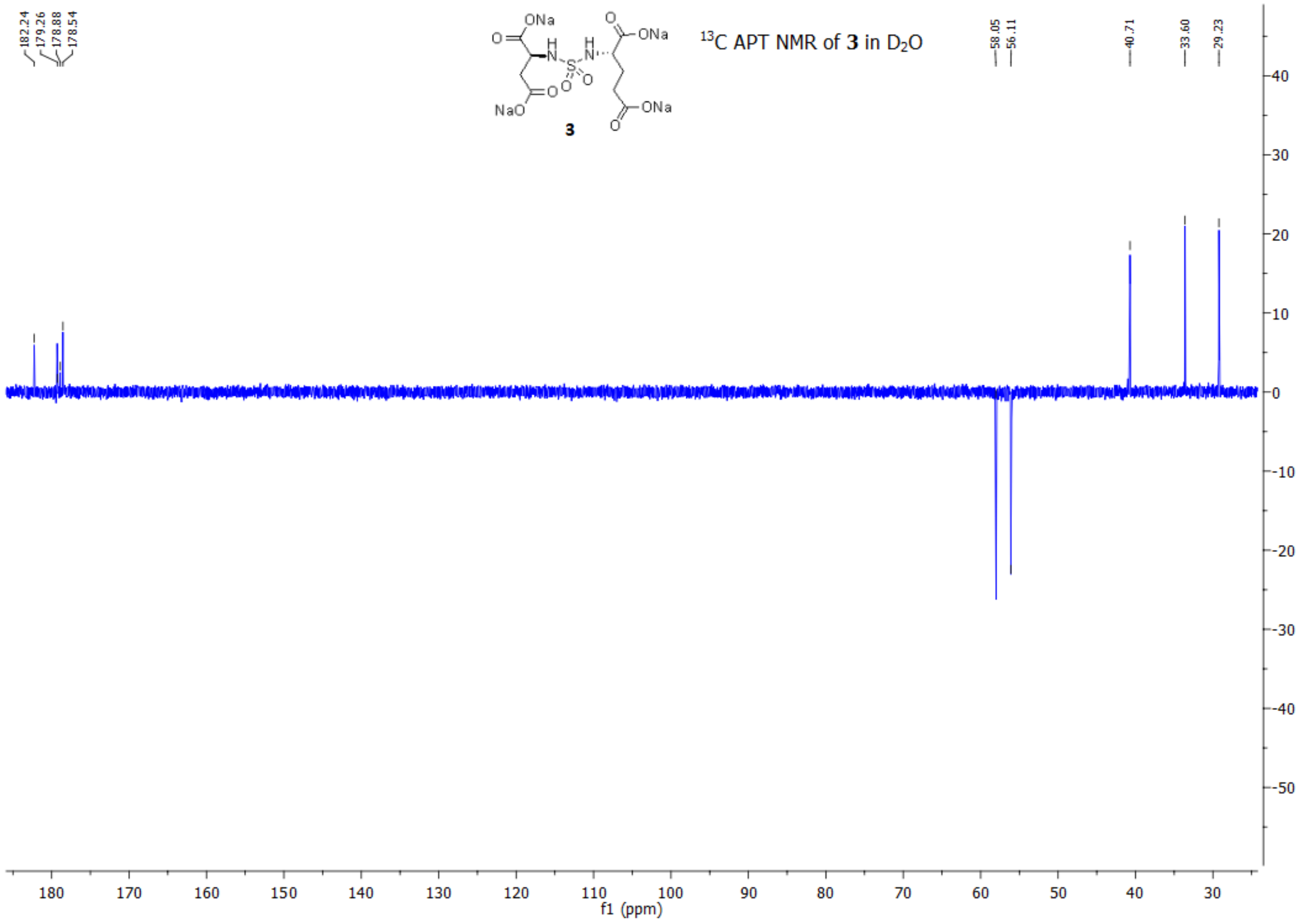

^{13}C APT NMR of **3** in D_2O
182.24
179.26
178.88
178.54
58.05
56.11
40.71
33.60
29.23
ONa
NaO
3
f1 (ppm)

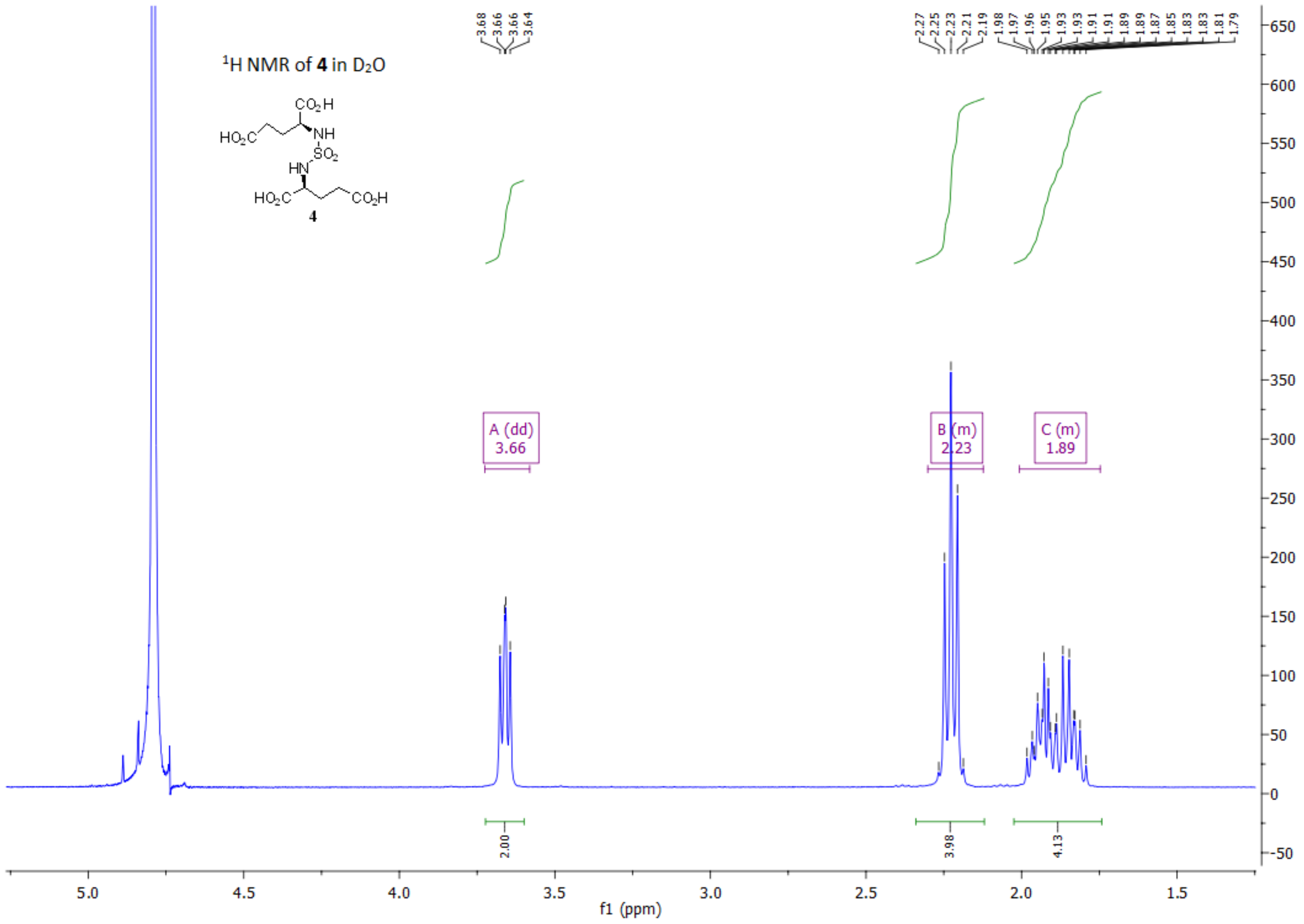

^{1}H NMR of **4** in D_2O
4
3.68 3.66 3.66 3.64
2.27 2.25 2.23 2.21 2.19 1.98 1.97 1.96 1.95 1.93 1.93 1.91 1.91 1.89 1.89 1.87 1.85 1.83 1.83 1.81 1.79
A (dd) 3.66
B (m) 2.23
C (m) 1.89
2.00
3.98
4.13
f1 (ppm)

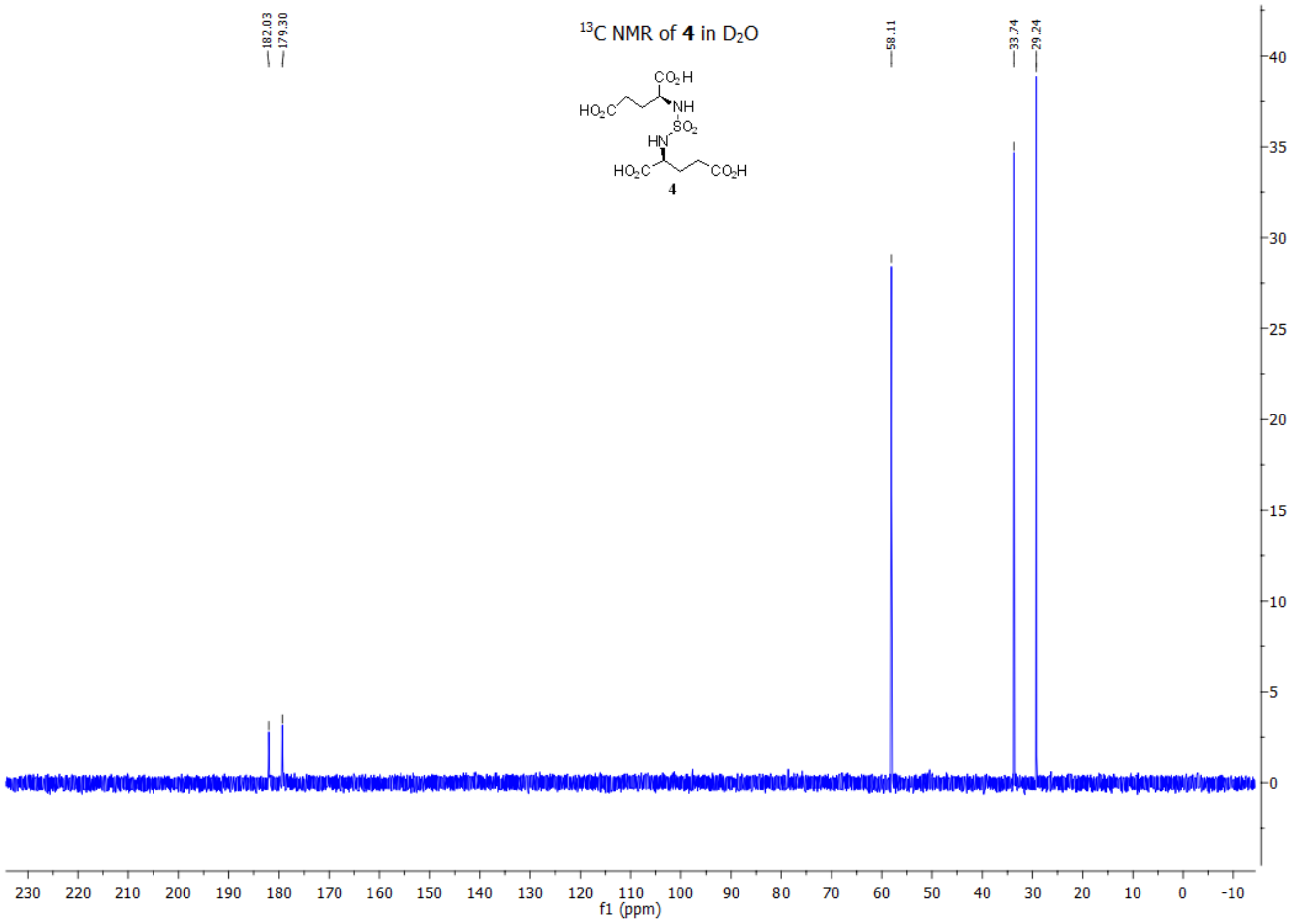

Fig. S3. Analytical data (NMR spectra) of the synthesized compounds.

Complex	E_{rel}, ep=4	E_{rel}, ep=8	E_{rel}, ep=20	E_{rel}, ep=40	E_{rel}, ep=78.4
NH-N	1.0	0.0	0.0	0.0	0.0
NH-O1	34.0	34.3	35.2	35.6	34.1
NH-O2	12.3	11.5	11.6	11.6	9.7
NH2-N	0.0	1.0	2.5	3.0	3.2
NH2-O1	9.9	12.0	14.1	14.9	14.8
NH2-O2	3.3	2.3	2.4	2.4	2.3

Table S1. The relative stability order of PSMA/9 complexes (structures are given in Fig. 3) calculated at the DFT-D3/TPSS/def2-TZVP level in different dielectric constants (ep= 4, 8, 20, 40 and 78.4)

Complex	E_{rel}, ep=4	E_{rel}, ep=8	E_{rel}, ep=20	E_{rel}, ep=40	E_{rel}, ep=78.4
PSMA/3-Water	0.0	0.0	0.0	0.0	0.0
PSMA/3-OH	40.5	33.7	28.8	27.1	10.6
**Complex**	**E_{rel}, ep=4**	**E_{rel}, ep=8**	**E_{rel}, ep=20**	**E_{rel}, ep=40**	**E_{rel}, ep=78.4**
PSMA/4-Water	0.0	0.0	0.0	0.0	0.0
PSMA/4-OH	52.0	44.3	38.9	36.9	36.0

Table S2. The relative stability order of PSMA/3 and PSMA/4 complexes (structures are given in Fig. 5) calculated at the DFT-D3/TPSS/def2-TZVP level in different dielectric constants (ep= 4, 8, 20, 40 and 78.4)
